# A neural basis for antagonistic control of feeding and compulsive behaviors

**DOI:** 10.1038/s41467-017-02534-9

**Published:** 2018-01-04

**Authors:** Leandra R. Mangieri, Yungang Lu, Yuanzhong Xu, Ryan M. Cassidy, Yong Xu, Benjamin R. Arenkiel, Qingchun Tong

**Affiliations:** 1 Brown Foundation Institute of Molecular Medicine of McGovern Medical School, University of Texas Health Science Center at Houston, Houston, TX 77030 USA; 2 Graduate Program in Neuroscience of MD Anderson Cancer Center UTHealth Graduate School of Biomedical Sciences, University of Texas Health Science Center at Houston, Houston, TX 77030 USA; 3 Department of Pediatrics, Children’s Nutrition Research Center, Baylor College of Medicine, One Baylor Plaza, Houston, TX 77030 USA; 4 Department of Molecular & Human Genetics, Baylor College of Medicine, Houston, TX 77030 USA; 5 Department of Neuroscience and Jan Duncan Neurological Research Institute, Texas Children’s Hospital, Houston, TX 77030 USA; 6 Department of Neurobiology and Anatomy of McGovern Medical School, University of Texas Health Science Center at Houston, Houston, TX 77030 USA

## Abstract

Abnormal feeding often co-exists with compulsive behaviors, but the underlying neural basis remains unknown. Excessive self-grooming in rodents is associated with compulsivity. Here, we show that optogenetically manipulating the activity of lateral hypothalamus (LH) projections targeting the paraventricular hypothalamus (PVH) differentially promotes either feeding or repetitive self-grooming. Whereas selective activation of GABAergic LH→PVH inputs induces feeding, activation of glutamatergic inputs promotes self-grooming. Strikingly, targeted stimulation of GABAergic LH→PVH leads to rapid and reversible transitions to feeding from induced intense self-grooming, while activating glutamatergic LH→PVH or PVH neurons causes rapid and reversible transitions to self-grooming from voracious feeding induced by fasting. Further, specific inhibition of either LH→PVH GABAergic action or PVH neurons reduces self-grooming induced by stress. Thus, we have uncovered a parallel LH→PVH projection circuit for antagonistic control of feeding and self-grooming through dynamic modulation of PVH neuron activity, revealing a common neural pathway that underlies feeding and compulsive behaviors.

## Abstract (web summary)

Feeding disorders are often comorbid with compulsive behaviours but the underlying neural circuits are not known. Here the authors demonstrate that glutamatergic and GABAergic projections from the LH to the PVH exert antagonistic effects on grooming and feeding via modulation of PVH neuron activity.

## Introduction

Feeding is essential for survival, and abnormal eating habits can manifest in the form of either uncontrolled hyperphagia or anorexia, leading to obesity or life-threatening nutrient insufficiency^1,2^. Although, it is well known that such feeding abnormalities severely impact the quality of life, the neural basis that underlies uncontrolled over-feeding or restrictive dieting remains elusive. Accumulating evidence supports the notion that feeding behavior reciprocally interacts with affective behaviors^1–3^. Overeating and obesity are often associated with an emotional state of increased impulsivity and positive reinforcement, similar to drug addiction^4,5^. Anorexia nervosa has several diagnostic subtypes associated with compulsive restriction, compulsive bingeing with purging, and compulsive hoarding^1,6^. Interestingly, it has been suggested that compulsive behaviors associated with anorexia nervosa may manifest as a way to relieve stress and anxiety^1,7^. In rodents, asocial repetitive self-grooming is a typical behavior that models compulsivity in neuropsychiatric diseases^8^. Recent observations^9^ suggest a provocative link between feeding disturbances and psychological dysregulation related to compulsivity, supporting a common neural pathway underlying maladaptive feeding and compulsive behaviors. However, despite extensive research on both feeding and psychiatric compulsivity, it is unclear whether a shared neural pathway regulates both behaviors.

The hypothalamus has been well established to regulate feeding behaviors. Discrete groups of neurons within the hypothalamus have been identified to be capable of sensing changes in various hormones or neuronal inputs in order to gauge nutritional status, and adjust food intake to maintain proper energy homeostasis^10^. The lateral hypothalamus (LH) functions as a hunger center, and modulates feeding and other homeostatic processes^11^. Current studies demonstrate that the LH contains two distinct groups of neurons that exert opposite actions on feeding regulation. GABAergic neurons have been implicated in driving feeding^12,13^, in part via local LH GABAergic projections to the ventral tegmental area or paraventricular hypothalamus (PVH)^13,14^. In contrast, LH glutamatergic neurons were shown to suppress feeding^15^, at least in part by their projections to the lateral habenula^16^. The physiologic implication of the contrasting effects on feeding behavior by LH GABAergic and glutamatergic neurons has remained unexplored.

The PVH, an important integration site, receives massive synaptic inputs from multiple brains areas, and plays an important role in feeding regulation^17^. The PVH receives melanocortin inputs from arcuate peptidergic POMC and AgRP neurons, which have been well characterized in their roles towards feeding regulation^18^. Recent studies demonstrated that the PVH also receives both GABAergic and glutamatergic inputs from the arcuate nucleus, thereby potently promoting and inhibiting food intake, respectively^19,20^. Our previous work supports a role for GABAergic inputs from LH to PVH in feeding promotion^13^. However, a role for glutamatergic projections from LH to PVH has not been characterized. Importantly, whether LH and PVH, the well-established brain regions for feeding regulation, directly modulate affective states such as compulsivity is unknown.

Here, we utilize transgenic mice that express cre recombinase from the pancreas-duodenum homeobox 1 promoter (*Pdx1-Cre*) to access mixed GABAergic and glutamatergic LH neurons^13^. We find that LH→PVH GABAergic projections promote feeding and suppress self-grooming, whereas glutamatergic projections promote excessive self-grooming and suppress feeding. Direct modulation of these behaviors is achieved through antagonistic control of PVH neuron activity. These findings provide a novel framework for understanding circuit-level mechanisms that link feeding and compulsive behaviors.

## Results

### LH→PVH projections directly synapse on a common subset of PVH neurons

**Fig. 1:**
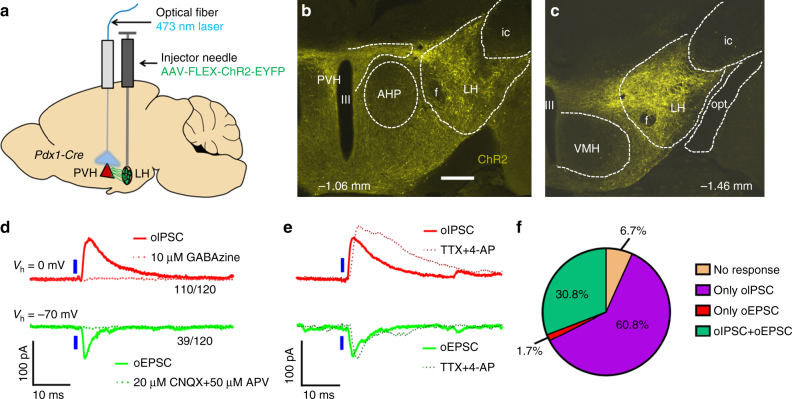
LH GABA and glutamate neurons send monosynaptic projections to a common subset of PVH neurons. **a** Experimental schematic. **b** Representative image showing ChR2-EYFP LH^*Pdx1*^ fibers in PVH. **c** ChR2 expression in LH^*Pdx1*^ neurons. Coordinates in **a**, **b** are anterior–posterior (AP) measurements relative to Bregma. III, 3rd ventricle; AHP, anterior hypothalamic area, posterior; f, fornix; ic, internal capsule; LH, lateral hypothalamus; opt, optic tract; PVH, paraventricular nucleus of the hypothalamus; Scale bar = 300 µm. **d** Electrophysiology traces from patch-clamp recordings showing optical-evoked inhibitory post-synaptic currents (oIPSCs, top) and optical-evoked excitatory post-synaptic currents (oEPSCs, bottom) in a PVH neuron held at the indicated potentials (*V*_h_) following 1-ms blue light pulse to LH^*Pdx1-ChR2*^ fibers. Currents can be blocked by ionotropic GABA or glutamate receptor blockers, GABAzine and CNQX+APV, respectively. **e** Effect of co-application of TTX and 4-AP in oIPSCs (top) and oEPSCs (bottom). **f** Pie chart showing percentage of PVH neurons receiving excitatory and/or inhibitory inputs from LH^*Pdx1-ChR2*^ neurons, as suggested by electrophysiology current responses to blue light

To selectively mark and manipulate LH neurons, we employed transgenic *Pdx1-Cre* mice. In this line, Cre is abundantly expressed in the LH, the dorsal medial hypothalamus (DMH), the Arc, and preoptic areas, but not in the PVH^21^. Within the LH, *Pdx1-Cre* neurons reside in medial-caudal regions, and are not present in rostral domains (Supplementary Fig. 1a–h). Regions of the LH known to express *Pdx1-Cre* were shown to contain GABAergic and glutamatergic neuron populations, which were largely segregated (Supplementary Fig. 1i–k). To investigate how LH neurons modulate PVH neurons, we first monitored monosynaptic projections from LH *Pdx1-Cre* (LH^*Pdx1*^) neurons to PVH neurons using channelrhodopsin 2 (ChR2)-assisted circuit mapping^22^. Towards this, we bilaterally delivered AAV-FLEX-ChR2-EYFP viral vectors to the LH in *Pdx1-Cre* mice (LH^*Pdx1-ChR2*^; Fig. 1a). 3 weeks post-injection, we identified ChR2-EYFP expressing neurons in the LH (Fig. 1c and Supplementary Fig. 2), and observed their associated projections in the PVH (Fig. 1b). Targeted photostimulation of LH fibers revealed both excitatory post-synaptic currents (oEPSCs) and inhibitory post-synaptic currents (oIPSCs) in PVH neurons in acute brain slices from LH^*Pdx1-ChR2*^ mice. To ensure that recorded responses were comparable between animals, we targeted neurons in the dorsal medial part of the PVH (spanning anterior-posterior Bregma coordinates, −1.06 mm to −1.22 mm), since this region contained abundant ChR2 expressing projections from LH^*Pdx1-ChR2*^ neurons (Fig. 1b). We found that > 91% of the recorded cells (110 out of 120) showed inhibitory responses, while < 33% (39 out of 120) showed excitatory responses (Fig. 1d). These photo-induced postsynaptic currents were blocked by bath-applied ionotropic GABA and glutamate receptor antagonists, respectively (Fig. 1d). Interestingly, the majority of neurons that showed light-evoked EPSCs also exhibited IPSCs (Fig. 1f). Both oEPSCs and oIPSCs in the PVH were resistant to bath-applied tetrodotoxin (TTX) and 4-aminopyridine (4-AP), known to block action potentials and inhibit network activity^23^, suggesting monosynaptic connectivity (Fig. 1e). Thus, LH→PVH glutamatergic and GABAergic inputs target a common subset of PVH neurons.

### Activation of LH→PVH fibers induces feeding and self-grooming

**Fig. 2:**
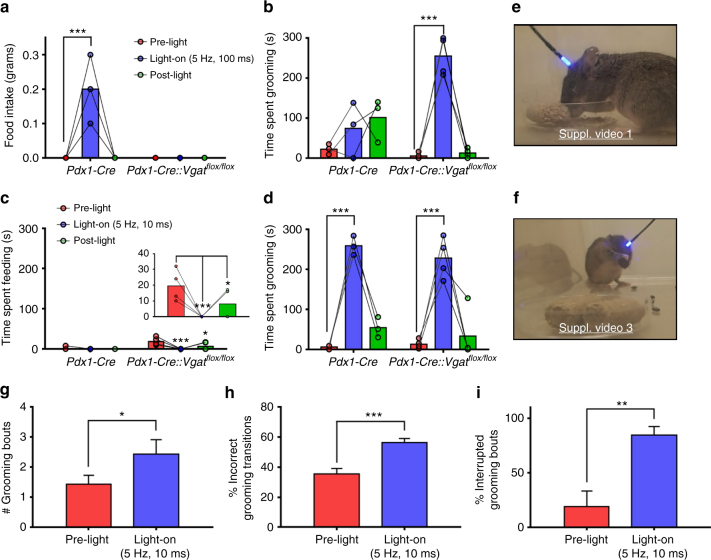
Optical activation of LH^*Pdx1*^→PVH projections differentially causes feeding and repetitive grooming behaviors, with the former requiring GABA release. Food intake (**a**) and grooming time (**b**) before (pre-light), during (light-on), and after (post-light) 5 Hz, 100 ms photostimulation of LH^*Pdx1-ChR2*^→PVH fibers in *Pdx1-Cre* and *Pdx1-Cre::Vgat* ^*flox/flox*^ mice. Feeding time (**c**) and grooming time (**d**) before, during, and after 5 Hz, 10 ms photostimulation of LH^*Pdx1-ChR2*^→PVH fibers in the same mice. Insert in **c** is expanded from *Pdx1-Cre::Vgat* ^*flox/flox*^ grooming data in the same figure. Snapshots taken from videos of *Pdx1-Cre* (**e**) and *Pdx1-Cre::Vgat* ^*flox/flox*^ (**f**) engaged in feeding and grooming behaviors, respectively, during light-on epoch (5 Hz, 100 ms) (Supplementary Videos 1 and 3). **g**–**i** Grooming microstructure characterization during pre-light and light-on (5 Hz, 10 ms) epochs. **a**–**d** and **g**–**i** Pre-light, light-on, and post-light epochs occurred consecutively and lasted 5 min each; *Pdx1-Cre*, *n* = 3 animals; *Pdx1-Cre::Vgat* ^*flox/flox*^, *n* = 4 animals. **a**–**d** Two-way repeated measures ANOVA followed by Dunnett’s multiple comparisons test. (**a** interaction F [2, 10] = 17.14, *P* = 0.0006; genotype F [1, 5] = 17.14, *P* = 0.0090; light, F [2, 10] = 17.14, *P* = 0.0006; subjects [matching] F [5, 10] = 1, *P* = 0.4651. *Pdx1-Cre* [pre-light vs. light-on] ***p < 0.0005. **b** interaction F [2, 10] = 15.77, *P* = 0.0008; genotype F [1, 5] = 6.342, *P* = 0.0533; light F [2, 10] = 19.48, *P* = 0.0004; subjects [matching] F [5, 10] = 0.2401, *P* = 0.9356. *Pdx1-Cre::Vgat* ^*flox/flox*^ [pre-light vs. light-on] ***p < 0.0005. **c** interaction F [2, 10] = 5.273, *P* = 0.0273; genotype F [1, 5] = 4.739, *P* = 0.0814; light F [2, 10] = 9.287, *P* = 0.0052; subjects (matching) F [5, 10] = 3.261, *P* = 0.0527. *Pdx1-Cre::Vgat* ^*flox/flox*^ [pre-light vs. light-on] ***p < 0.0005; [pre-light vs. post-light *p < 0.05. **d** interaction F [2, 10] = 0.3491, *P* = 0.7136; genotype F [1, 5] = 1.443, *P* = 0.2834; light F [2, 10] = 58.37, *P* < 0.0001; subjects [matching] F [5, 10] = 0.4241, *P* = 0.8220. *Pdx1-Cre* and *Pdx1-Cre::Vgat* ^*flox/flox*^ [pre-light vs. light-on] ***p < 0.0005. **g**–**i** two-tailed, paired Student's *t*-tests; data are presented as mean ± s.e.m. **g** *t* = 3.24, df = 6, *P = 0.0177. **h** *t* = 7.996, *df* = 6, ***P = 0.0002. **i** *t* = 4.174, *df* = 6, **P = 0.0059)

Similar to our previous study^13^, we activated LH^*Pdx1-ChR2*^→PVH terminal fibers using optic fibers implanted above the PVH (Fig. 1a and Supplementary Fig. 2a, b). A stimulation protocol of blue 473 nm light pulsed at constant 5 Hz and 100 ms pulse-width duration caused voracious feeding in *Pdx1-Cre* mice (Fig. 2a, e and Supplementary Video 1). However, conditional knockout mice lacking the vesicular GABA transporter (*Vgat*, which is required for presynaptic GABA release) and expressing ChR2 in LH (*Pdx1-Cre::Vgat* ^*flox/flox*^ mice) showed no alteration in feeding behavior during light stimulation (Fig. 2a and Supplementary Fig. 2c). Electrophysiological recordings in PVH brain slices of *Pdx1-Cre::Vgat* ^*flox/flox*^ mice confirmed selective disruption of GABA release from LH^*Pdx1-ChR2*^ fibers to PVH neurons (Supplementary Fig. 3a, b). These results are consistent with our previous findings that GABA release from LH→PVH terminals promotes feeding behavior^13^. Using these models, we also assayed behavioral responses during photostimulation of LH→PVH terminals when food was removed, and observed that most of *Pdx1-Cre* mice tested exhibited aberrant licking behavior (Supplementary Video 2), which was never observed in *Pdx1-Cre::Vgat* ^*flox/flox*^ mice. LH→PVH evoked behavioral responses were comparable between unilaterally and bilaterally injected mice with ChR2; thus, data from these two groups were combined and presented herein.

Interestingly, we noted that in a 5 min period following 5 Hz, 100 ms light stimulation, *Pdx1-Cre* mice tended to exhibit self-grooming behavior (Fig. 2b). Strikingly, during the 5 min light-on period, *Pdx1-Cre::Vgat* ^*flox/flox*^ mice exhibited extensive repetitive self-grooming behavior (Fig. 2b, f and Supplementary Video 3). These data suggest that GABA release in LH→PVH suppresses evoked grooming behavior. To gauge the activation threshold of LH→PVH fibers in driving feeding behavior, we evaluated multiple stimulation protocols for the ability to elicit grooming vs. feeding behavior. We found that, in *Pdx1-Cre* mice, photostimulation with 5 Hz, 10 ms pulses failed to induce any feeding (Fig. 2c), and instead induced intense self-grooming (Fig. 2d). Similarly, the same stimulation regime did not elicit feeding in the *Pdx1-Cre::Vgat* ^*flox/flox*^ mice, but instead suppressed the time spent feeding (Fig. 2c), and led to repetitive self-grooming that was indistinguishable from that in *Pdx1-Cre* mice (Fig. 2d). Multiple trials revealed that 5 Hz, 100 ms light pulses consistently induced feeding responses, whereas 5 Hz, 10 ms pulses consistently induced self-grooming in *Pdx1-Cre* animals. Thus, we used these two protocols to differentially induce feeding and self-grooming in this study. As a control, *Pdx1-Cre* mice that received cre-dependent GFP virus (opsin-negative vectors) to LH did not exhibit elevated feeding or grooming behaviors upon PVH illumination (Supplementary Fig. 4).

Stressful and anxiety-provoking situations increase self-grooming behavior and alter the natural progression of grooming transitions, which normally proceed in an uninterrupted cephalocaudal direction^24^. With this in mind, we investigated whether light-evoked self-grooming resulted in changes in stress-related grooming “microstructure.” Using a grooming analysis algorithm^25^ we analyzed the number of grooming bouts, incorrect grooming transitions, and interrupted grooming bouts, and found that all these components of grooming behavior were significantly increased (Fig. 2g–i), suggesting that LH→PVH stimulation induced grooming is stress-related in nature.

### Glutamate release from LH→PVH fibers in self-grooming behavior

The rapid onset of self-grooming behavior by LH→PVH activation is independent of GABA release, suggesting that glutamate may mediate this effect. To explore this, we used *Pdx1-Cre::Vglut2* ^*flox/flox*^ mice (*Vglut2*, also named Slc17a6, is required for presynaptic glutamate release in most hypothalamic neurons). I*n situ* hybridization data showed that the number of *Vglut2*-expressing neurons in the LH was dramatically reduced in *Pdx1-Cre::Vglut2* ^*flox/flox*^ mice compared to controls (Supplementary Fig. 6a, b), suggesting that a significant portion of LH^*Pdx1*^ neurons are glutamatergic. To evaluate glutamate release from LH^*Pdx1*^ neurons via *Vglut2* deletion, we recorded oIPSCs and oEPSCs in PVH neurons following blue light stimulation of LH^*Pdx1-ChR2*^ fibers in *Pdx1-Cre::Vglut2* ^*flox/flox*^ mice. 0/12 PVH neurons exhibited oEPSCs in response to blue light (Supplementary Fig. 3c). Concurrently, 8 out of 12 PVH neurons showed oIPSCs (Supplementary Fig. 3d), demonstrating that deletion of *Vglut2* in *Pdx1-Cre* neurons leads to selective loss of glutamate release from LH→PVH terminals while GABAergic neurotransmission remains intact.

**Fig. 3:**
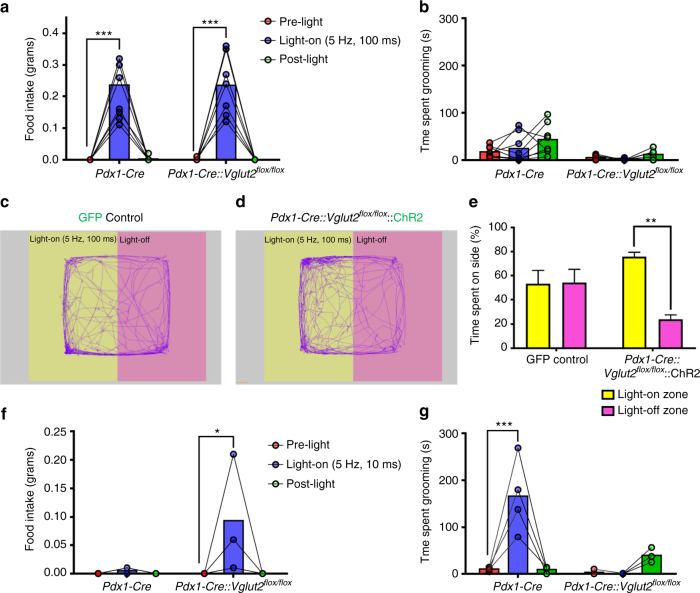
LH^*Pdx1*^→PVH evoked grooming requires glutamate release, and activation of non-glutamatergic fibers promotes behavioral approach. Food intake (**a**) and grooming time (**b**) before (pre-light), during (light-on), and after (post-light) 5 Hz, 100 ms photostimulation of LH^*Pdx1-ChR2*^→PVH fibers in *Pdx1-Cre* (*n* = 8) and *Pdx1-Cre::Vglut2* ^*flox/flox*^ (*n* = 7) mice. Real-time place preference assay (RTPP) during optical stimulation of LH^*Pdx1*^→PVH in GFP control (*n* = 6) and *Pdx1-Cre::Vglut2* ^*flox/flox*^::ChR2 mice (*n* = 4). **c**, **d** Representative tracks tracing locomotion; **e** Percentage time spent on each side. **e** Two-way ANOVA followed by Sidak’s multiple comparisons test (interaction F [1, 16] = 7.192, *P* = 0.0164; viral injection F [1, 16] = 0.1407, *P* = 0.7125; light zone F [1, 16] = 6.612, *P* = 0.0205. *Pdx1-Cre::Vglut2* ^*flox/flox*^::ChR2 (Light-off zone vs. Light-on zone) **p < 0.005. Data presented as mean ± s.e.m. Food intake **(f)** and grooming time **(g)** before, during, and after 5 Hz, 10 ms photostimulation of LH^*Pdx1-ChR2*^→PVH fibers in *Pdx1-Cre* (*n* = 4) and *Pdx1-Cre::Vglut2* ^*flox/flox*^ (*n* = 3) mice. **a**, **b, f** and **g** Two-way repeated measures ANOVA followed by Dunnett’s multiple comparisons test. (**a** interaction F [2, 26] = 0.003466, *P* = 0.9965; genotype F [1, 13] = 0.000817, *P* = 0.9776; light F [2, 26] = 66.15, *P* < 0.0001, subjects (matching) F [13, 26] = 0.9467, *P* = 0.5233. *Pdx1-Cre* and *Pdx1-Cre::Vglut2* ^*flox/flox*^ [pre-light vs. light-on] ***p < 0.0005. **b** interaction F [2, 26] = 1.065, *P* = 0.3593; genotype F [1, 13] = 10.98, *P* = 0.0056; light F [2, 26] = 3.622, *P* = 0.0409; subjects (matching) F [13, 26] = 1.472, *P* = 0.1942. **f** interaction F [2, 10] = 3.073, *P* = 0.0912; genotype F [1, 5] = 3.073, *P* = 0.1400; light F [2, 10] = 3.808, *P* = 0.0590; subjects (matching) F [5, 10] = 1, *P* = 0.4651. *Pdx1-Cre::Vglut2* ^*flox/flox*^ (pre-light vs. light-on) *p < 0.05. **g** interaction F [2, 10] = 14.45, *P* = 0.0011; genotype F [1, 5] = 8.36, *P* = 0.0341; light F [2, 10] = 8.57, *P* = 0.0068; subjects [matching] F [5, 10] = 1.078, *P* = 0.4280. *Pdx1-Cre* (pre-light vs. light-on) ***p < 0.0005)

To test the behavioral effects of LH→PVH terminal stimulation, *Pdx1-Cre* and *Pdx1-Cre::Vglut2* ^*flox/flox*^ mice received LH injections of AAV-FLEX-ChR2-EYFP vectors and optic fiber implants over PVH (Supplementary Fig. 5). In vivo 5 Hz, 100 ms light stimulation of LH^*Pdx1-ChR2*^ fibers in the PVH led to comparable ravenous feeding in both *Pdx1-Cre* and *Pdx1-Cre::Vglut2* ^*flox/flox*^ mice (Fig. 3a). Similar to the previous findings (Fig. 2b), light stimulation elicited mild self-grooming in some *Pdx1-Cre* mice, but no grooming in *Pdx1-Cre::Vglut2* ^*flox/flox*^ mice (Fig. 3b). When stimulated with 5 Hz, 10 ms, *Pdx1-Cre* mice showed no changes in feeding (Fig. 3f), but exhibited intense self-grooming (Fig. 3g). On the other hand, *Pdx1-Cre::Vglut2* ^*flox/flox*^ mice exhibited feeding (Fig. 3f), but no grooming behavior (Fig. 3g). In the absence of food, 5 Hz, 100 ms light stimulation elicited extensive licking behavior in *Pdx1-Cre::Vglut2* ^*flox/flox*^ mice, which differed from the combined licking and grooming behavior seen in *Pdx1-Cre* mice (Supplementary Fig. 6c). When presented with a piece of square bedding in the absence of food, some *Pdx1-Cre::Vglut2* ^*flox/flox*^ mice violently chewed and ripped the bedding (Supplementary Fig. 6d). These findings support our conclusion that LH→PVH GABAergic activity strongly promotes the drive to feed and suppresses grooming, while LH→PVH glutamatergic activity promotes self-grooming.

We next probed the valence of LH→PVH activity in *Pdx1-Cre::Vglut2* ^*flox/flox*^ mice using a real-time place preference assay (RTPP)^16^. Mice previously unexposed to the test were placed in a large test chamber with two equal zones: a light-off zone paired with no light stimulation, and a light-on zone paired with 5 Hz, 100 ms blue light stimulation. Control mice that received **AAV-FLEX-GFP** injections into the LH and optic fibers implants above PVH did not display significant preference for either the light-paired or light-unpaired side of the chamber (Fig. 3c, e). In contrast, *Pdx1-Cre::Vglut2* ^*flox/flox*^ mice expressing ChR2 in LH^*Pdx1*^ and with optic fibers implanted above PVH, significantly preferred the light-paired side of the chamber over the light-off zone (Fig. 3d, e). Together, these data suggest that activation of GABAergic LH→PVH circuit produces positive valence.

### PVH neurons mediate feeding and self-grooming behavior

Activation of remote sites through collateral fiber activation remains a caveat of targeting projections for stimulation with optogenetics^26^. Thus, to determine if photostimulation of LH^*Pdx1-ChR2*^ fibers in the PVH activated additional LH collaterals in non-PVH sites through antidromic action, we crossed *Pdx1-Cre* mice with *Sim1-Cre::γ2* ^*flox/flox*^ mice^27^, in which the GABA-A receptor γ2 subunit, an essential subunit for GABA-A receptor function, is deleted in *Sim1*-expressing PVH neurons. The resulting *Pdx1-Cre::Sim1-Cre::γ2* ^*flox/flox*^ mice lacked γ2 subunit in both *Pdx1-Cre* and *Sim1-Cre* neurons^13^. We used *Pdx1-Cre::γ2* ^*flox/flox*^ mice as a control group. Surprisingly, spike firing rates of PVH neurons between *Sim1-Cre* and *Sim1-Cre::γ2* ^*flox/flox*^ mice were similar (Supplementary Fig. 7). Further whole cell recordings showed that γ2 deletion in *Sim1-Cre* PVH (PVH^*Sim1*^) neurons led to a reduction in evoked IPSCs as well as evoked EPSCs (data not shown), suggesting that the lack of change in PVH^*Sim1*^ neuron activity with γ2 deletion is due to a compensatory reduction of glutamatergic inputs.

**Fig. 4:**
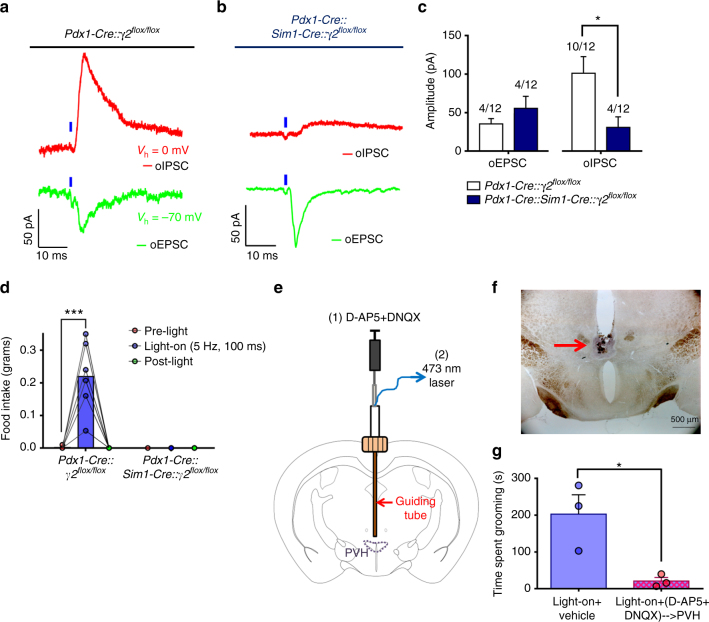
GABA-A and ionotropic glutamate receptor activation in PVH are required for LH^*Pdx1-ChR2*^→PVH evoked feeding and grooming, respectively. **a** Inhibitory (oIPSC, top) and excitatory (oEPSC, bottom) post-synaptic current responses evoked by 1-ms blue light pulse (blue tick) in a PVH neuron of a *Pdx1-Cre::γ2* ^*flox/flox*^ mouse (control). **b** oIPSC (top) and oEPSC (bottom) responses to 1-ms blue light pulse in a PVH neuron of a *Pdx1-Cre::Sim1-Cre:: γ2* ^*flox/flox*^ mouse (double-cre knockout). **c** Quantification of current amplitude in control and double-cre knockout mice. Number of cells showing current response out of total number of cells recorded is shown above bars. Data are presented as mean ± s.e.m. (two-tailed Mann–Whitney test; median for control mice = 101.3, *n* = 10; median for double-cre knockout mice = 19.8, *n* = 4; *U* = 4; *P = 0.0240). **d** Food intake before (pre-light), during (light-on), and after (post-light) 5 Hz, 100 ms photostimulation of LH^*Pdx1-ChR2*^→PVH fibers in control (*n* = 7) and double-cre knockout mice (*n* = 6). Two-way repeated measures ANOVA followed by Dunnett’s multiple comparisons test. (interaction F [2, 22] = 28.85, *P* < 0.0001; genotype F [1, 11] = 29.11, *P* = 0.0002; light F [2, 22] = 28.85, *P* < 0.0001; subjects [matching] F [11, 22] = 1.01, *P* = 0.4689. *Pdx1-Cre::γ2* ^*flox/flox*^ [pre-light vs. light-on], *** *p* < 0.0005). **e** Schematic illustrating custom-made guide cannula allowing for interchangeable fluid and optical delivery to PVH. **f** Approximate fluid injection location in PVH area is shown by microinjection of blue ink before sacrifice. Scale bar = 500 µm. **g** Time spent grooming during 5 min of 5 Hz, 10–50 ms photostimulation of LH^*Pdx1-ChR2*^→PVH following vehicle or D-AP5 + DNQX microinjection to PVH (two-tailed ratio paired t-test; t = 6.862 df = 2; *P = 0.0206; *n* = 3)

We then performed whole-cell recordings on PVH neurons following photostimulation of LH^*Pdx1-ChR2*^ fibers. Whereas light evoked comparable monosynaptic EPSCs in both *Pdx1-Cre::γ2* ^*flox/flox*^ mice and *Sim1-Cre::Pdx1-Cre::γ2* ^*flox/flox*^ mice (Fig. 4a, b), oIPSCs displayed significantly reduced amplitudes in *Sim1-Cre::Pdx1-Cre::γ2* ^*flox/flox*^ mice compared to that of *Pdx1-Cre::γ2* ^*flox/flox*^ mice (Fig. 4b, c). In addition, a smaller fraction of neurons displayed oIPSCs in *Sim1-Cre::Pdx1-Cre::γ2* ^*flox/flox*^ compared to *Pdx1-Cre::γ2* ^*flox/flox*^ mice (Fig. 4c). However, the fraction of neurons that displayed oEPSCs was similar between groups (Fig. 4c). Behaviorally, light stimulation of LH^*Pdx1-ChR2*^→PVH fibers in control *Pdx1-Cre::γ2* ^*flox/flox*^ mice induced voracious feeding (Fig. 4d and Supplementary Fig. 8) similar to that observed in *Pdx1-Cre* mice. In contrast, the same photostimulation paradigm failed to induce any feeding behavior in *Sim1-Cre::Pdx1-Cre::γ2* ^*flox/flox*^ mice (Fig. 4d and Supplementary Fig. 8).

It is possible that non-PVH, *Sim1-Cre* positive brain regions expressing γ2 mediated the light-evoked feeding behavior via collateral activation and consequent release of GABA to these regions. Thus, we examined if LH *Pdx1-Cre* fibers project to *Sim1* neurons in non-PVH brain sites by implementing conditional viral vectors that contained a cre-dependent synaptophysin-EGFP reporter^28^ injected into the LH of *Pdx1-Cre::Sim1-Cre::Ai9* mice (Supplementary Fig. 9a and c). Synaptophysin-EGFP fluorescence localizes in presynaptic terminals, thus serving as an effective anterograde tracer. In these animals, abundant EGFP expression was noted in the caudal portions of the PVH, as expected (Supplementary Fig. 9b). None of the dense, non-PVH *Sim1-Cre* expressing brain regions, including the nucleus of lateral olfactory tract, medial amygdala, and premammillary nucleus, showed obvious EGFP expression (Supplementary Fig. 9d–f), suggesting that non-PVH, *Sim1-Cre*-expressing brain regions do not contribute to the evoked feeding behavior. Altogether, these data show that PVH neurons mediate the avid feeding response observed with local stimulation of LH→PVH fibers.

To test whether PVH neurons mediate self-grooming induced by photostimulation of LH^*Pdx1-ChR2*^→PVH fibers, we employed a custom-made guide cannula that allowed simultaneous delivery of light and local drug infusion to targeted PVH areas (Fig. 4e). As glutamate release contributes significantly to the evoked self-grooming behaviors (Fig. 3g), we examined the effect of local infusion of glutamate receptor antagonists (D-AP5+DNQX) to the PVH preceding photostimulation (Fig. 4f). Drug infusion significantly reduced self-grooming behavior induced by photostimulation of LH^*Pdx1-ChR2*^→PVH fibers, compared to the saline infused condition (Fig. 4g). Collectively, these studies demonstrate that PVH neurons mediate light-evoked self-grooming behavior by receiving LH→PVH glutamatergic input.

### LH→PVH projections in physiologic feeding and grooming

**Fig. 5:**
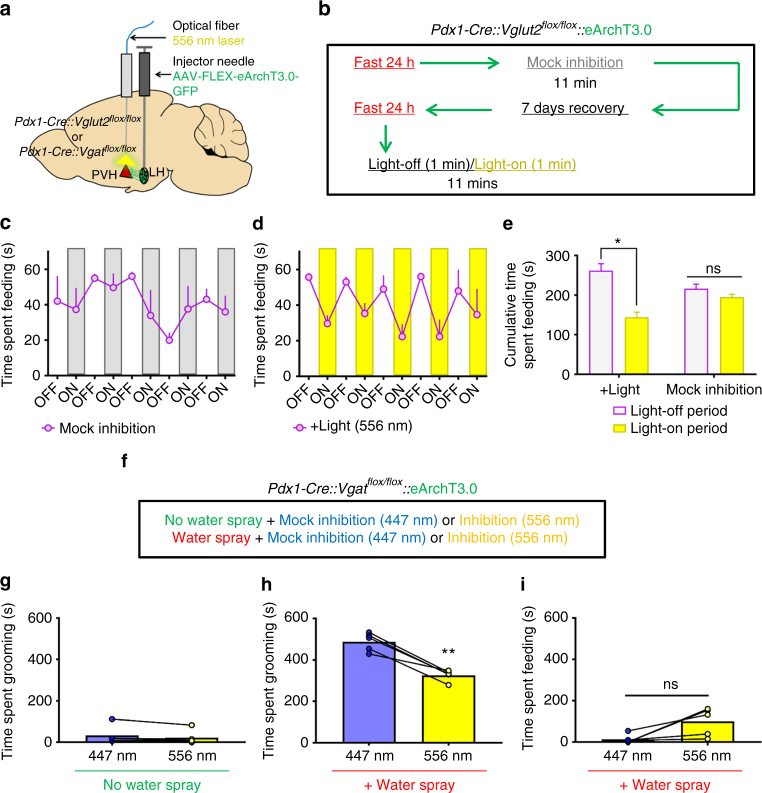
Inhibition of GABAergic LH^*Pdx1*^→PVH fibers reduces feeding after a fast, and inhibition of glutamatergic fibers reduces water-spray induced grooming. **a** Experimental schematic. **b** Experimental protocol used to test fast-refeeding in *Pdx1-Cre::Vglut2* ^*flox/flox*^::eArchT3.0 mice under mock or 556 nm light inhibition. **c** Time spent refeeding after a fast during mock inhibition trial. **d** Time spent re-feeding after a fast under + Light trial, whereby 556 nm constant light was applied every other minute during 10 consecutive minutes. **e** Comparison of cumulative time spent feeding during light-off vs. light-on periods during + Light and Mock inhibition trials. *n* = 3 animals. Two-way repeated measures ANOVA followed by Sidak’s multiple comparisons test; light group F (1, 2) = 0.01648, *P* = 0.9096; light period F (1, 2) = 45.77, *P* = 0.0212; interaction light group X light period F (1, 2) = 35.58, *P* = 0.0270. Light-off vs. Light-on period (+Light group) *p < 0.05. Data are presented as mean ± s.e.m. **f** Experimental protocol used to test water spray induced grooming in *Pdx1-Cre::Vgat* ^*flox/flox*^::eArchT3.0 mice under mock (447 nm) or 556 nm light inhibition. **g** Time spent grooming during mock and 556 nm inhibition when no water spray was delivered. **h** Time spent grooming during mock and 556 nm inhibition when water spray was delivered to induce grooming. **i** Simultaneous time spent feeding during water spray trials. **g**–**i** Two-tailed paired Student's *t* test; *n* = 5; **g** *t* = 1.935, *df* = 4, *p* = 0.1250; **h** *t* = 7.779, *df* = 4, **p = 0.0015; **i** *t* = 2.731, *df* = 4, *p* = 0.0524 (ns, not significant)

To examine the physiological relevance of LH→PVH projections in feeding, we next tested whether increased LH→PVH GABAergic action contributes to fast-refeeding. Towards this, we delivered conditional viral vectors encoding the light-driven outward proton pump eArchT3.0 bilaterally into LH of *Pdx1-Cre::Vglut2* ^*flox/flox*^ mice and implanted optic fibers above the PVH (Fig. 5a). This allowed silencing of neurotransmitter release from LH presynaptic terminals upon yellow light illumination in the PVH area^12^. Strong GFP fluorescence was observed in both LH neurons and LH→PVH fibers in mice that received **AAV-FLEX-eArchT3.0-GFP** injections to LH (LH^*Pdx1-eArchT3*.0^; Supplementary Fig. 10a). To verify that illumination of LH^*Pdx1-eArchT3.0*^ fibers reduced GABA release, we recorded oIPSCs in PVH neurons of *Pdx1-Cre* mice following photostimulation of LH^*Pdx1-ChR2-eArchT3.0*^ fibers that simultaneously expressed eArchT3.0 and ChR2 (see Methods). Whereas blue light (473 nm) reliably evoked oIPSCs in PVH neurons, yellow light (556 nm) greatly diminished blue-light evoked oIPSCs (Supplementary Fig. 10b). This effect was reversed after removal of yellow light (Supplementary Fig. 10b). These data showed that eArchT3.0 activation effectively inhibited presynaptic release of GABA from LH→PVH terminals. To examine the feeding effects of light-mediated inhibition of GABAergic LH→PVH terminals, we fasted *Pdx1-Cre::Vglut2* ^*flox/flox*^ expressing eArchT3.0 in LH^*Pdx1*^ and tested them the following day with the protocol described in Fig. 5b. Mice continuously engaged in food consumption during 10 min of fast-refeeding during mock inhibition trials (Fig. 5c, e). During +Light trials, where yellow light illuminated above PVH every other minute (constant 556 nm), the cumulative time spent eating was significantly reduced during light-on, compared to the light-off period (Fig. 5d, e). At the same time, grooming behavior was unaltered by light condition (Suppl. Fig. 10c). These results showed that ongoing GABA transmission in LH→PVH circuit contributes significantly to physiological feeding behavior. To test whether ongoing glutamatergic transmission from LH→PVH is required for stress-induced grooming, we used *Pdx1-Cre::Vgat* ^*flox/flox*^ mice expressing eArchT3.0 in LH^*Pdx1*^ for photoinhibition experiments outlined in Fig. 5f (also see Suppl. Fig. 10d–e). Baseline grooming levels during 556 nm light inhibition was unaltered compared to mock inhibition trials with blue light (Fig. 5g). In contrast, elevated grooming caused by water spraying the mice, a procedure known to elevate stress-related grooming^29^, was significantly attenuated with 556 nm vs. mock inhibition (Fig. 5h). At the same time, feeding behavior was not significantly affected by light condition following water spray (Fig. 5i).

### Antagonistic control of feeding and grooming by LH→PVH projections

**Fig. 6:**
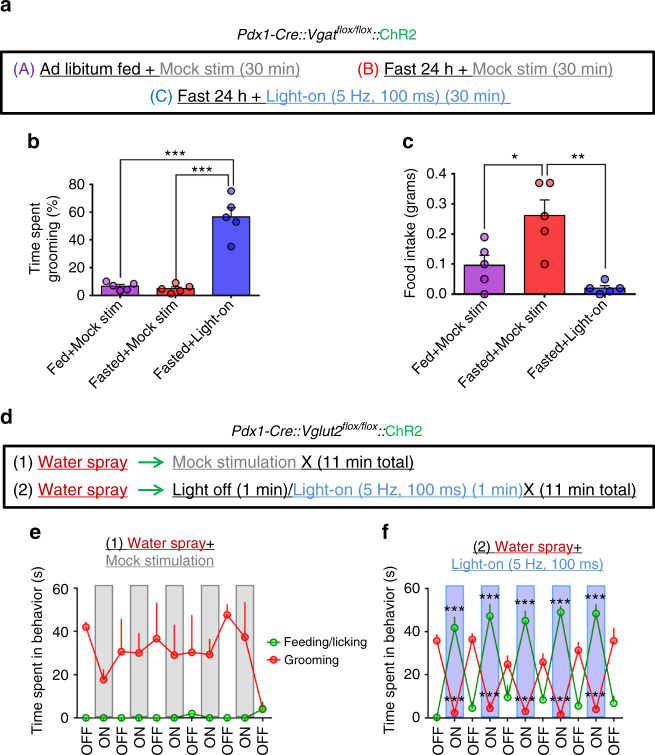
Antagonistic control of feeding and self-grooming by GABAergic and glutamatergic LHPdx1→PVH fibers. **a** Experimental design for testing effects of glutamatergic LH^*Pdx1-ChR2*^→PVH fiber photostimulation on grooming and refeeding after a fast. **b** The percentage of time spent grooming during three 30 min trials. **c** Food intake during the same three trials. **b**, **c** Data are presented as mean ± s.e.m.; *n* = 5 animals; repeated measures ANOVA followed by Tukey’s multiple comparisons. (**b** Trial condition F [2, 8] = 55.01, *P* < 0.0001; subjects F [4, 8] = 0.9764, *P* = 0.4714. Fasted+light vs. Fed+Mock stim and Fasted + Mock stim ***p < 0.0005. **c** Trial condition F [2, 8] = 12.37, *P* = 0.0036; subjects F [4, 8] = 1.062, *P* = 0.4344. Fed + Mock stim vs. Fasted + Mock stim *p < 0.05; Fasted + Mock stim vs. Fasted + light **p < 0.005.) **d** Experimental protocol to test whether photostimulation of GABAergic LH^*Pdx1-ChR2*^ →PVH fibers interferes with grooming induced by water spray. **e** Time spent engaged in grooming or feeding/licking behaviors during mock stimulation (*n* = 3). **f** Time spent grooming or feeding/licking during light-on condition, where 5 Hz, 100 ms light pulses were applied every other minute (*n* = 5). **e**, **f** Data are presented as mean ± s.e.m.; two-way repeated measures ANOVA followed by Dunnett’s multiple comparisons test. (**e** interaction F [10, 40] = 1.1, *P* = 0.3854; light F [10, 40] = 0.8106, *P* = 0.6199; behavior F [1, 4] = 231.6, *P* = 0.0001; subjects (matching) F [4, 40] = 0.2938, *P* = 0.8803. Grooming [first light-off vs. last light-off] *p < 0.05; **f** interaction F [10, 80] = 78.48, *P* < 0.0001; light F [10, 80] = 2.864, *P* = 0.0042; behavior F [1, 8] = 2.74, *P* = 0.1365; subjects (matching) F [8, 80] = 7.308, *P* < 0.0001. Feeding/licking (first light off vs. On-1, On-2, On-3, On-4, On-5) ***p < 0.0005; Grooming (first light off vs. On-1, On-2, On-3, On-4, On-5) ***p < 0.0005)

We next sought to determine if activity level of GABAergic or glutamatergic action in this circuit could drive competition between the two behaviors. To probe whether activation of the grooming component (i.e., glutamatergic transmission) competes with fast-refeeding, we fasted *Pdx1-Cre::Vgat* ^*flox/flox*^ mice expressing ChR2 in LH^*Pdx1*^ neurons (Supplementary Fig. 11a–d), and re-fed them for 30 min under either mock or blue light stimulation of LH ^*Pdx1-ChR2*^→PVH fibers (Fig. 6a). Fasted mice with mock stimulation (Fasted+Mock stim) showed a significant increase in food intake compared to that in the Fed+Mock stimulation condition (Fig. 6c). However, the time spent grooming between these two conditions were similar, and constituted < 7% of the total testing time (Fig. 6b). On the other hand, activation of LH^*Pdx1-ChR2*^→PVH fibers with 5 Hz, 100 ms blue light pulses during the fasted state (Fasted + Light-on) led to a significant increase in time spent grooming (56.6 ± 6.58 percent time spent grooming ± s.e.m.; Fig. 6a), and was accompanied by a corresponding decrease in food consumption (0.02 ± 0.01 grams ± s.e.m.) compared to the Fasted + Mock stim condition (0.26 ± 0.05 grams ± s.e.m.; Fig. 6c). These data suggest that fast-refeeding can be antagonized by self-grooming behavior induced by activation of LH→PVH glutamatergic inputs.

To genetically dissect the effects of grooming during GABAergic LH→PVH stimulation, we used *Pdx1-Cre::Vglut2* ^*flox/flox*^ mice that received ChR2 injections to LH and optic fibers implanted above PVH. We water sprayed the mice to evoke extensive self-grooming. After water spray, mice were placed in a bare cage for 11 min under mock stimulation conditions (Fig. 6d). Water spray reliably induced approximately 10 min of continuous grooming behavior, and had no effect on feeding related behaviors (Fig. 6e). In a separate trial where mice were water sprayed and then paired with photostimulation of LH^*Pdx1-ChR2*^→PVH (continuous 5 Hz, 100 ms, 473 nm applied every other minute), grooming behavior was dramatically suppressed, and was accompanied with a concomitant elevation in feeding behavior (Fig. 6f). Strikingly, the light effect on grooming and feeding behaviors was rapidly reversed during the light-off epochs, such that mice immediately stopped eating, and reverted back to grooming (Fig. 6f and Supplementary Video 4). Of note, the rapid elevation in grooming during the light-off periods was not due to post-light effects (Supplementary Fig. 11e). Together, these data show that activation of GABAergic LH→PVH fibers antagonizes stress-related grooming and promotes feeding behavior.

### Activation of PVH neurons on self-grooming versus feeding

**Fig. 7:**
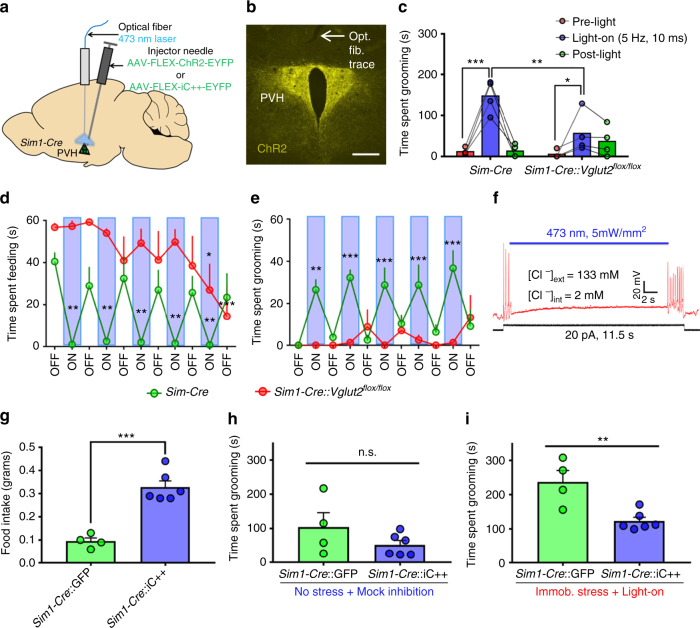
Photostimulation of PVH^*Sim1*^ neurons induces grooming behavior and competes with fast-refeeding, and inhibition increases feeding and reduces stress-induced grooming. **a** Experimental schematic. **b** ChR2-EYFP expression in PVH^*Sim1*^ neurons and optical fiber implantation (opt. fib. trace, arrow) above PVH. Scale bar = 300 µm. **c** Grooming time before (pre-light), during (light-on), and after (post-light) 5 Hz, 10 ms photostimulation of PVH^*Sim1-ChR2*^ neurons in *Sim1-Cre* (*n* = 4) and *Sim1-Cre::Vglut2* ^*flox/flox*^ (*n* = 4) mice. Two-way repeated measures ANOVA followed by Dunnett’s multiple comparisons test (interaction F [2, 12] = 10.77, *P* = 0.0021; genotype F [1, 6] = 2.265, *P* = 0.1831; light F [2, 12] = 30.2, *P* < 0.0001; subjects (matching) F [6, 12] = 2.514, *P* = 0.0821. *Sim1-Cre* [pre-light vs. light-on] ***p < 0.0005; *Sim1-Cre::Vglut2* ^*flox/flox*^ [pre-light vs. light-on] *p < 0.05; Sidak’s multiple comparisons: *Sim1-Cre* vs. *Sim1-Cre::Vglut2* ^*flox/flox*^ [light-on] **p < 0.005). **d** Time spent feeding after a fast during alternating, 1 min-light off/on events in *Sim1-Cre* (*n* = 4) and *Sim1-Cre::Vglut2* ^*flox/flox*^ (*n* = 4) mice (light-on = 5 Hz, 10 ms). **e** Time spent grooming in the same experiment. **d**, **e** Data are presented as mean ± s.e.m. Two-way repeated measures ANOVA followed by Dunnett’s multiple comparisons test **d**, interaction F [10, 60] = 4.594, *P* < 0.0001; light F [10, 60] = 4.265, *P* = 0.0002; genotype F [1, 6] = 15.95, *P* = 0.0072; subjects (matching) F [6, 60] = 5.389, *P* = 0.0002. *Sim1-Cre* [first light-off vs. On-1, On-2, On-3, On-4, On-5] **p < 0.005; *Sim1-Cre::Vglut2* ^*flox/flox*^ [first light-off vs. last light-on] *p < 0.05; (first light-off vs. last light-off) ***p < 0.0005. **e**, interaction F [10, 60] = 5.418, *P* < 0.0001; light F [10, 60] = 3.68, *P* = 0.0007; genotype F [1, 6] = 12, *P* = 0.0134; subjects (matching) F [6, 60] = 3.74, *P* = 0.0032. *Sim1-Cre* (first light-off vs. On-1) **p < 0.005; (first light-off vs. On-2, On-3, On-4, On-5) ***p < 0.0005). **f** Silencing PVH^*Sim1-iC++*^ neurons with blue light blocks action potentials induced by depolarizing current injection. **g** 30 min food intake during optical inhibition of PVH with blue light in *Sim1-Cre*::GFP control and iC++ mice. **h** Time spent grooming during 15 min of mock inhibition following no acute stress in the same mice. **i** Time spent grooming during 15 min of blue-light inhibition following 10 min of immobilization stress in the same mice. **g**–**i** Two-tailed Student's *t*-tests. **g** *t* = 6.715, *df* = 8, ***p = 0.0002; **h** *t* = 1.415, *df* = 8, *p* = 0.1948; **i** *t* = 3.855, *df* = 8, **p = 0.0048

The above data suggest that the activity level of PVH neurons determines feeding vs. self-grooming behaviors. To directly test this, we bilaterally delivered **AAV-FLEX-ChR2-EYFP** to PVH of *Sim1-Cre* mice (PVH^*Sim1-ChR2*^; Fig. 7a, b and Supplementary Fig. 12). Whole cell electrophysiological recordings in acute brain slices showed that blue light pulses reliably excited PVH^*Sim1-ChR2*^ neurons (Supplementary Fig. 12f). Our previous studies showed that glutamate release from PVH was essential in mediating PVH function in body weight regulation^30^. As such, we also included *Sim1-Cre::Vglut2* ^*flox/flox*^ mice in the following optogenetic studies. Recapitulating results from LH^*Pdx1-ChR2*^→PVH fiber photostimulation, 5 Hz, 10 ms blue light stimulation in PVH^*Sim1-ChR2*^ neurons evoked repetitive self-grooming behavior in *Sim1-Cre* mice (Fig. 7c). However, *Sim1-Cre::Vglut2* ^*flox/flox*^ mice exhibited significant blunting of grooming behavior with the same light stimulation (Fig. 7c). The elevation in grooming behavior induced by photostimulation was absent in *Sim1-Cre* mice receiving **AAV-FLEX-GFP** viral injections to PVH (Supplementary Fig. 13). To examine the effect of PVH neuron activation on feeding and grooming behaviors, both groups of mice were fasted for 24 h, then refed for 11 min with blue light stimulation (5 Hz, 10 ms, 473 nm, every other minute). *Sim1-Cre* mice showed persistent repetitive self-grooming and suppressed feeding during light-on periods. During light-off periods, the mice rapidly switched to feeding and displayed minimal grooming (Fig. 7d, e and Supplementary Video 5). In contrast, *Sim1-Cre::Vglut2* ^*flox/flox*^ mice did not have light-associated grooming behavior and fed regardless of light condition (Fig. 7d, e and Supplementary Video 6). These results suggest that an increased level of PVH neuron activity promotes self-grooming and inhibits feeding in a glutamate release-dependent manner. We next probed whether optogenetically inhibiting PVH^*Sim1*^ neurons would affect feeding and grooming. To this end, *Sim1-Cre* mice were injected with **AAV-FLEX-iC++-EYFP** vectors to allow expression of the inhibitory channel iC++^31^ in PVH neurons (Supplementary Fig. 12g). Patch clamp experiments showed that blue light inhibited firing of PVH neurons in *Sim1-Cre*::iC++ mice (Fig. 7f). Acute inhibition of PVH^*Sim1*^ caused a significant increase in food intake compared with GFP-injected controls (Fig. 7g). Furthermore, elevated grooming induced by immobilization stress was significantly attenuated in *Sim1-Cre*::iC++, compared to GFP controls during blue-light inhibition (Fig. 7i). The shortened grooming response in iC++ mice was not due to differences in baseline levels of grooming compared to GFP controls (Fig. 7h). These data support that overall activity of PVH neurons significantly participates in normal feeding and stress-related grooming behaviors, and further reinforce the idea that LH→PVH projections regulate both feeding and self-grooming behaviors by modulating PVH neuron activity.

In addition to PVH, LH neurons are known to project to lateral habenula and ventral tegmental area (VTA)^14,16^. To examine whether PVH-projecting LH neurons also send collaterals to these brain regions, we used a previously established pseudorabies viral tracing method^32^ to label PVH-projecting LH neurons with GFP. While we observed some GFP-labeled fibers in the dorsomedial hypothalamus and LH, none was found in lateral habenula or VTA (Supplementary Fig. 14), suggesting that PVH-projecting LH neurons do not send significant collaterals to lateral habenula or VTA.

## Discussion

The etiology of compulsive behaviors and their association with feeding abnormalities remains poorly understood^1,8^. Previous studies on the pathophysiology of compulsivity and related disorders mainly focus on forebrain regions^1,8,9,33^. Our results showed that parallel GABAergic and glutamatergic LH→PVH projections can differentially promote feeding vs. repetitive self-grooming by modulating PVH neuron activity. While simultaneous activation of both projections induced mixed feeding and self-grooming, selective activation of one blocked the behavior mediated by the other, demonstrating an antagonistic regulation of these two behaviors by LH→PVH projections. Thus, our study defines previously unknown hypothalamic circuit capable of promoting two competing behaviors, i.e., feeding and self-grooming behavior, and provides a framework for the LH→PVH pathway as a potentially important brain mechanism underlying eating disorders associated with compulsivity.

The use of *Pdx1-Cre* mouse line in our study offers a number of advantages over *Vglut2-Cre* and Vgat-*Cre*. This unique model allowed us to gain access to both LH glutamate and GABA neurons, thus allowing simultaneous activation of LH→PVH GABAergic and glutamatergic terminals. With this dual activation, we identified a common subset of PVH neurons that receive monosynaptic glutamatergic and GABA inputs from LH neurons. The model also allowed the ability to show differential behavioral outcomes with optogenetic activation of the mixed glutamatergic and GABAergic LH→PVH terminals using differing strengths of light stimulation, suggesting neurotransmitter competition for behavior within the LH→PVH circuit. The combinational use of *Pdx1-Cre* with floxed *Vglut2* and *Vgat* alleles also allows specific disruption of glutamate and GABA release and demonstration of the role of the individual neurotransmitters without competing effects from the other. Furthermore, use of *Pdx1-Cre* facilitated genetic confirmation of functional PVH contribution, via *Sim1-Cre* mediated γ2 deletion, in mediating LH→PVH in feeding. Notably, the baseline readings of feeding and self-grooming, two major behaviors that were monitored in this study, were not altered in these knockout mice. Thus, the impact of potential developmental alterations associated with gene knockouts, if it exists, minimally affects our data interpretation.

We discovered that activation of glutamatergic LH→PVH terminals caused extensive, repetitive self-grooming, suggesting a high level of compulsivity. The induced self-grooming behavior was shown to be stress-related, which is consistent with previous observations on the role of LH orexin and leptin responsive neurons in increasing stress levels^34^. Remarkably, the evoked repetitive self-grooming behavior consistently overrode extreme hunger-induced feeding following 24 h fasting. Thus, LH→PVH glutamatergic projection activation produced a phenotype analogous to the maladaptive behaviors often described in human anorexia nervosa, which consist of obsessive thoughts surrounding body weight, compulsive exercise, and extreme eating aversion associated with long-term self-induced starvation^35–38^. Importantly, inhibition of both glutamatergic transmission from LH to PVH and PVH neurons reduced extensive self-grooming behavior induced by stress, suggesting an involvement of activation of PVH neurons by increased glutamate release from the LH in the etiology of stress-related self-grooming. In humans, many forms of compulsive behaviors, including eating disorders, co-exist with higher levels of stress^24,39^. Thus, chronic activation of LH→PVH glutamatergic projections may potentially serve as a model for compulsive anorexia in humans.

In addition to feeding behavior, LH→PVH GABAergic projections also promoted feeding-related behaviors in the absence of food, such as licking and chewing up bedding, suggesting an increased level of impulsivity and an uncontrolled drive to eat. Similar aberrant feeding-associated behaviors were also observed during activation of LH→VTA GABAergic projections and LH GABAergic neurons^14,40^. This feeding behavior is distinct from that mediated by AgRP neuron activation, which shows a specific effect toward foraging and feeding, and is not associated with aggressive licking or chewing^41–43^. Our data revealed that mice displayed behavioral approach to a side of a test chamber paired with GABAergic LH→PVH activation. In line with this, recent results show that AgRP GABAergic inputs to both LH and PVH are also associated with positive reinforcement^44^. The positive valence associated with LH→PVH-induced feeding may escalate the drive for feeding and its related behaviors. Thus, chronic activation of LH→PVH GABAergic projections may contribute to conditions characterized by uncontrolled overeating.

One striking finding in this study is that LH→PVH glutamatergic projections were in parallel with LH→PVH GABAergic projections. Our findings suggest that short duration stimulation of mixed glutamatergic and GABAergic LH→PVH projections may preferentially activate glutamatergic components and increase PVH neuron excitability, leading to self-grooming, while long duration stimulation may cause a net GABAergic action and reduce PVH neuron activity, leading to feeding. Importantly, we found that the net effect of changing PVH neuron activity swiftly changed behavioral outcomes. Elevating PVH neuron activity by photostimulating PVH^*Sim1-ChR2*^ neurons caused repetitive self-grooming and inhibited feeding within seconds even during extreme hunger induced by fasting. Similarly, photostimulation of glutamatergic LH→PVH projections abruptly suppressed feeding after a fast, and induced self-grooming. In contrast, inhibiting PVH neuron activity caused the opposite effect, causing increased feeding and attenuation of stress-induced grooming. Consistently, activation of GABAergic projections in LH→PVH circuit potently disrupted repetitive grooming induced by water spray, and promoted feeding, an effect that was rapidly reversed upon photostimulation termination. Thus, acute changes in PVH neuron activity in response to dynamic GABAergic and glutamatergic inputs give rise to self-grooming or feeding. Unbalanced glutamatergic and GABAergic transmission to PVH may underlie feeding disturbances associated with compulsivity. As LH→PVH-evoked feeding behavior is associated with positive reinforcement, and the evoked grooming is associated with stress, a typical negative reinforcement, these results suggest a scalable regulation of PVH neuron activity on emotional states.

Given the rapid switch between feeding and self-grooming by changing LH→PVH GABAergic and glutamatergic activity, or by acutely activating or inhibiting PVH neurons, it is likely that a shared PVH neuron population mediates the two behaviors. Supporting this, our data suggest that LH^*Pdx1*^ neurons send monosynaptic GABAergic and glutamatergic projections to a common subset of PVH neurons. Provided the established role of PVH melanocortin receptor 4 (MC4R)-expressing neurons in feeding regulation^18,23^ these neurons present themselves as a candidate. Notably, MC4R and SAPAP3 double deletion rescues both hyperphagia from MC4R deficiency, and excessive self-grooming from SAPAP3 deficiency^9^, raising a possibility that PVH MC4R-neurons contribute to this effect. Activation of PVH thyroxine releasing hormone (TRH) neurons promotes feeding but does not lead to self-grooming^45^, an opposite effect that would be expected from LH→PVH glutamatergic activation. Thus, TRH neurons are an unlikely candidate. Notably, PVH neurons expressing corticotropin releasing hormones (CRH) might contribute to self-grooming behavior^46^. Further studies are required to identify the exact subset(s) of PVH neurons that dynamically regulate feeding and self-grooming.

Feeding is essential for survival, and animals respond to fasting by consuming a large amount of food immediately when it becomes available to offset the energy deficit occurred during fasting periods. Despite the key importance of fast-refeeding responses, the neural pathway essential for this life-preserving behavior remains unclear. Thus far, AgRP neurons represent the prevailing group of neurons established in mediating fast-refeeding responses^47,48^. Our results showed that optogenetic inhibition of LH→PVH GABAergic terminals strongly inhibited fast-refeeding and was robust and repeatable, suggesting that an active release of GABA in PVH mediates ongoing fast-refeeding. Thus, we identified LH GABAergic neurons as a novel population of neurons, alongside AgRP neurons, that are capable of governing fast-refeeding behavior. Supporting this, our previous studies showed that loss of GABA release from *Pdx1-Cre* neurons reduced fast-refeeding^27^.

In summary, using a combination of optogenetics, mouse genetics, behavioral assays, and pharmacology, we identified a single LH→PVH projection with GABAergic and glutamatergic components that promote feeding and self-grooming behaviors, respectively. Importantly, dynamic PVH neuron activity changes regulated by glutamatergic and GABAergic inputs induced rapid and reversible transitions between feeding and self-grooming, raising an interesting possibility that defective LH→PVH activity may contribute to eating disorders associated with compulsive behaviors.

## Methods

### Animal care

Mice were housed at 21–22 °C with a 12 h light/12 h dark cycle with standard pellet chow and water provided ad libitum unless otherwise noted for fasting experiments. Animal care and procedures were approved by the University of Texas Health Science Center at Houston Institutional Animal Care and Use Committee. *Pdx1-Cre* and *Sim1-Cre* mice were described previously^21,49^. Mice with the conditional allele of vesicular GABA transporter (*Vgat*, also named *Slc32a1*), and mice with the conditional allele of vesicular glutamate transporter 2 (*Vglut2*, also named *Slc17a6*) were reported previously^50,51^. Mice containing GABA-A receptor 2 subunit (γ2) conditional allele were also reported previously^52^. The following breeding pairs were maintained to generate study subjects: 1*. Pdx1* ^*cre/+*^ *::Vgat* ^*flox/+*^ X *Vgat* ^*flox/flox*^; 2. *Pdx1* ^*cre/+*^ *::Vglut2* ^*flox/+*^ X *Vglut2* ^*flox/flox*^; 3. *Pdx1* ^*cre/+*^ *::γ2* ^*flox/flox*^ X *Sim1* ^*cre/+*^ *:: γ2* ^*flox/flox*^; 4. *Sim1* ^*cre/+*^ *::Vglut2* ^*flox/+*^ X *Vglut2* ^*flox/flox*^. Additionally, in most breeding pairs, either male or female breeders (or both) contained the Ai9 reporter gene to allow RFP expression in the presence of cre recombination^53^. Both male and female mice were used as study subjects. Mice used in experiments were obtained from multiple litters and were at least 6 weeks old.

### Surgeries and viral constructs

Stereotaxic surgeries to deliver viral constructs and for optical fiber implantation were performed as previously described^54^. Briefly, mice were anesthetized with a ketamine/xylazine cocktail (100 mg/kg and 10 mg/kg, respectively), and their heads affixed to a stereotaxic apparatus. Viral vectors were delivered through a 0.5 µL syringe (Neuros Model 7000.5 KH, point style 3; Hamilton, Reno, NV, USA) mounted on a motorized stereotaxic injector (Quintessential Stereotaxic Injector; Stoelting, Wood Dale, IL, USA) at a rate of 40 nL/min. Viral preparations were titered at ~10^12^ particles/mL. Viral delivery was targeted to the LH (200–400 nL/side; anteroposterior (AP): −1.0 mm; mediolateral (ML):±1.1 to 1.3 mm; dorsoventral (DV): −5.0 mm) or PVH (65–150 nL/side; AP: −0.5 to −0.6 mm; ML:±0.2 to 0.3 mm; DV: −5.0 mm). Uncleaved fiber optic cannulae (Ø1.25 mm Stainless Ferrule, Ø200 µm Core, 0.39 NA; ThorLabs, Newton, New Jersey, USA) were precut to 4.5–4.8 mm and implanted above the PVH (AP: −0.5 to −0.6 mm; ML: 0 mm). Optical fibers were secured on the head with dental cement.

For blue-light dependent activation of LH→PVH fibers and PVH neurons, *AAV-EF1α-DIO-hChR2(H134R)-EYFP-WPRE-hGHpA* (Addgene, plasmid number 20298) serotype 2 out of 9^29^ or *AAV-EF1α-DIO-hChR2(H134R)-EYFP-WPRE-pA* serotype 2 out of 5 (University of North Carolina Vector Core, Chapel Hill, NC, USA) (general name used throughout text for ChR2 virus: AAV-FLEX-ChR2-EYFP) was injected unilaterally or bilaterally into the LH of *Pdx1-Cre, Pdx1-Cre::Vgat* ^*flox/flox*^ *, Pdx1-Cre::Vglut2* ^*flox/flox*^ *, Pdx1-Cre:: γ2* ^*flox/flox*^, and *Pdx1-Cre::Sim1-Cre:: γ2* ^*flox/flox*^ or PVH of *Sim1-Cre* and *Sim1-Cre::Vglut2* ^*flox/flox*^ mice. For light-dependent inhibition of LH→PVH fibers, *AAV-CAG-DIO-eArchT3.0-EGFP* (general name used throughout text for eArchT3.0 virus: AAV-FLEX-eArchT3.0-[E]GFP)serotype 2 out of 9 was delivered bilaterally into LH of *Pdx1-Cre::Vglut2* ^*flox/flox*^ and *Pdx1-Cre::Vgat* ^*flox/flox*^ mice. For PVH^*Sim1*^ inhibition experiments, *AAV-EF1α-DIO-iC*++*-EYFP* (University of North Carolina Vector Core, Chapel Hill, NC, USA) (general name used throughout text for iC++ virus: AAV-FLEX-iC++ -EYFP) was injected bilaterally into the PVH of *Sim1-Cre* mice. *AAV-EF1α-DIO-EGFP* (general name used throughout text for GFP virus: AAV-FLEX-[E]GFP) serotype DJ8 was injected into the LH and PVH of *Pdx1-Cre* mice and *Sim1-Cre* mice, respectively, and used as an opsin-negative control for behavioral experiments. For electrophysiology recordings testing suppression of light-evoked current response, *Pdx1-Cre* mice received a cocktail injection containing 50:50 mix of cre-dependent ChR2+eArchT3.0 viruses to the LH. For anterograde viral tracing, *AAV-EF1α-FLEX-Syn::EGFP-WPRE-hGHpA*, serotype DJ/8^28^ (general name used for Synaptophysin virus: AAV-FLEX-Synaptophysin-GFP) was injected unilaterally into LH of *Pdx1-Cre::Sim1-Cre::Ai9* mice. Cre-dependent viruses cited from^28^, as well as eArchT3.0 and GFP were made in-house by Dr. Ben Arenkiel’s lab.

### Brain slice electrophysiological recordings

Coronal brain slices (250–300 μm) containing the PVH from mice that had received stereotaxic injections of AAV-FLEX-ChR2-YFP or AAV-FLEX-ChR2-YFP+AAV-FLEX-eArchT3.0-GFP to LH or PVH at least 3 weeks prior to the recording were cut in ice-cold artificial cerebrospinal fluid (aCSF) containing the following (in mM): 125 NaCl, 2.5 KCl, 1 MgCl_2_, 2 CaCl_2_, 1.25 NaH_2_PO_4_, 25 NaHCO_3_, and 11 d-glucose bubbling with 95% O_2_/5% CO_2_. Slices containing the PVH were immediately transferred to a holding chamber and submerged in oxygenated aCSF. Slices were maintained for recovery for at least 1 h at 32–34 °C before transferring to a recording chamber. Individual slices were transferred to a recording chamber mounted on an upright microscope (Olympus BX51WI) and continuously superfused (2 ml/min) with ACSF warmed to 32–34 °C by passing it through a feedback-controlled in-line heater (TC-324B; Warner Instruments). Cells were visualized through a 40X water-immersion objective with differential interference contrast (DIC) optics and infrared illumination. Whole cell voltage-clamp recordings were made from neurons within the sub-regions of the PVH that showed the highest density of ChR2-eYFP + axonal fibers. Patch pipettes (3–5 MΩ) were filled with a Cs^+^-based low Cl^−^ internal solution containing (in mM) 135 CsMeSO_3_, 10 HEPES, 1 EGTA, 3.3 QX-314, 4 Mg-ATP, 0.3 Na_2_-GTP, 8 Na_2_-Phosphocreatine (pH 7.3 adjusted with CsOH; 295 mOsm) for voltage-clamp. For current-clamp recordings, pipettes were filled with a K^+^-based low Cl^−^ internal solution containing (in mM) 145 KGlu, 10 HEPES, 0.2 EGTA, 1 MgCl_2_,4 Mg-ATP, 0.3 Na_2_-GTP, 10 Na_2_-Phosphocreatine (pH 7.3 adjusted with KOH; 295 mOsm). Membrane potentials were corrected for ~10 mV liquid junction potential. To activate ChR2 or ChR2 + eArchT3.0-expressing fibers from LH or ChR2-expressing neurons in PVH, light from a 447 nm or 473 nm laser (Opto Engine LLC, Midvale, UT, USA) was focused on the area of the recorded PVH neuron to produce spot illumination through optic fiber. Brief pulses of light (blue light (1–2 ms) and/or yellow light (556 nm, 200 ms); 1–2 mW/mm^2^) were delivered at the recording site at 10–15 s intervals under control of the acquisition software. GABAzine (10 µM) or CNQX+APV (20 µM and 50 µM) drugs (Abcam) were bath-applied to block GABA-A receptors or AMPA, kainate, and NMDA receptors, respectively, during voltage-clamp recordings of photostimulation-induced inhibitory or excitatory current responses. TTX (0.5 μM; Alomone labs, Jerusalem, Israel), and 4-AP (100 μM; ACROS Organics, Fisher Scientific, Pittsburgh, PA, USA) were bath-applied during voltage-clamp recordings of photostimulation-induced inhibitory and excitatory current responses to block action potentials and inhibit network activity. Following established protocols for loose-patch recordings in voltage clamp mode^55^, pipettes (2–3 MΩ) were filled with ACSF and the seal resistance was kept at 20–100 MΩ. The holding potential was routinely monitored and adjusted to maintain a holding current close to 0 pA in order to avoid changing the membrane potential of the cell being recorded. To activate iC^++^-expressing fibers^31^, blue light (473 nm, 5 mW/mm^2^, 10 s) was applied during AP firing induced by current injection of 10–30 pA, at 30 s intervals intervals under control of the acquisition software.

### Behavioral experiments

Behavioral testing was conducted during the light cycle following a minimum 3 week recovery period post-surgery. For in vivo photostimulation/inhibition experiments, an integrated rotary joint patch cable connected the ferrule end of optic fiber cannula with a Ø1.25 mm ferrule end of the patch cable via a mating ceramic sleeve (ThorLabs, Newton, New Jersey, USA). At the other end of the rotary joint, an FC/PC connector was connected to a 447 nm, 473 nm or 556 nm diode-pumped solid state (DPSS) laser (Opto Engine LLC, Midvale, Utah, USA). Light pulses were controlled by Master-8 pulse stimulator (A.M.P.I., Jerusalem, Israel). For Real Time Place Preference Assays, a commutator (rotary joint; Doric, Québec, Canada) was attached to a patch cable via FC/PC adapter. The patch cable was then attached to the optic fiber cannula ferrule end via a ceramic mating sleeve. Another patch cable containing FC/PC connections at both ends allowed the connection between the commutator and the laser, which was controlled by the Master-8 pulse stimulator. During testing, mice were placed in a fresh cage with no bedding. For RTPP assays, mice were placed into a clean testing chamber that was wiped down with 70% isopropyl alcohol between tests.

### Feeding and grooming assays

For optogenetic stimulation-feeding/grooming experiments, mice were ad libitum fed prior to testing (excluding competition experiments; see below). Mice underwent 15-min trials consisting of three consecutive 5-min epochs (pre-light, light-on, and post-light). During pre-light and post-light periods, the laser was turned off. During the light-on period, blue light (473 nm, ~5–10 mW/mm^2^) was pulsed at 5 Hz, with each pulse-width duration lasting 10 or 100 ms. Food intake was measured and recorded after the completion of each epoch during the 15-min trial. A video was recorded for each trial. Trials were repeated for each mouse at least three times on separate days to verify repeatability. An observer blind to the experimental conditions watched the videos and manually calculated the time spent grooming (see paragraph below), feeding, and/or licking with a stopwatch. Feeding time was counted when mice were actively engaged in biting, chewing, swallowing, or licking food. Licking time in the absence of food was counted when the tongue moved across the surface of the cage floors and walls.

For grooming quantification, an observer blind to genotype condition watched the 15-min videos and manually quantified the time spent grooming during each epoch with a stop watch. Grooming time was counted when the mouse engaged in forlimb paw strokes made near the nose, eyes, and head and during paw, body, tail, and genital licking. Rodent grooming is typically classified as a stereotypical chain of events that progresses from paw and nose grooming, to face grooming, to head grooming, and finally to body grooming^24^. To assess whether grooming induced by light activation of LH^*Pdx-ChR2*^→PVH fibers results in abnormal patterning of grooming events, an observer watched 5 Hz, 10 ms videos of *Pdx1-Cre* and *Pdx1-Cre::Vgat* ^*flox/flox*^ mice and quantified the number of grooming bouts, bout interruptions, and changes in grooming transitions using a grooming analysis algorithm described in ref. ^25^ during pre-light and light-on (5 Hz, 10 ms) conditions. To analyze small changes in grooming patterns, the observer watched videos on Quick Time Player (Apple), which easily allows for manual manipulation of video speed and pausing.

### Real time place preference (RTPP)

*Pdx1-Cre::Vglut2* ^*flox/flox*^ and *Pdx1-Cre*-GFP controls, injected with cre-dependent ChR2 or GFP viruses, respectively, and implanted with optic fibers above PVH, were placed in a clean 45 × 45 × 50 cm^3^ chamber equipped with a camera mounted on top of the chamber and optical fiber patch cable attached to a commutator. Prior to starting experiments, the patch cable was attached to optic fiber ferrule end of the mouse’s cannula. At the start of the experiment, mice were placed in the light-off zone, in which no light was applied. Then, for 20 min, the mice were allowed to freely roam the enclosure, which was divided into two equal zones containing the light-off zone and a light-on zone, in which 5 Hz, 100 ms (473 nm, ~5–10 mW/mm^2^) light pulses were delivered. The side paired with photostimulation was counterbalanced between mice. Tracking data, including time spent in each zone, were collected and analyzed by Ethovision XT software (version 11.5; Noldus, Wageningen, Netherlands). Preference for one of either side was determined by comparing the percentage of time spent in each zone.

### Ionotropic GluR blockade experiment

Custom-made guide cannulae (Doric, Québec, Canada) allowing for interchangeable fluid and light delivery were implanted above PVH in *Pdx1-Cre* mice containing ChR2 in LH^*Pdx1*^ neurons. Prior to testing, the dummy cannula was removed from the guiding cannula and the fluid-delivery cannula was inserted via a screw-on top mechanism. Either 100 nL vehicle (15% sterile DMSO in 0.9% saline) or 100 nL drugs (Tocris) containing 50 nL (61 mM D-AP5 solution in saline) + 50 nL (24 mM DNQX solution in 25–30% DMSO) were delivered via syringe (5 µL, Model 75 RN SYR, Small Removable NDL, 26 s ga, 2 in, point style 2; Hamilton, Reno, NV, USA). A plastic tube (RenaSil Silicone Rubber Tubing, .025 OD X .012 ID; Braintree Scientific, INC, Braintree, MA, USA) joined the syringe to the fluid-delivery cannula, and vehicle or drugs were manually infused at a rate of 100 nL/min. To prevent backflow of fluid, the fluid-delivery cannula was left screwed-on for an additional 2–3 min after infusion. Thereafter, the fluid-delivery cannula was screwed-off and the optical-fiber cannula was screwed on and attached to a fiber optic patch cable for light delivery and subsequent behavioral testing. Light was pulsed at 5 Hz, 10–50 ms (473 nm, 2–6 mW/mm^2^) for a 5-min trial, in which a video was recorded and later quantified for time spent grooming. Vehicle and drug trials were performed on separate days, and order of trial condition was randomized. Pulse duration for each mouse was determined by the lowest duration (starting at 10 ms) that would induce the maximal amount of grooming without inducing feeding.

### Inhibition experiments

*Pdx1-Cre::Vglut2* ^*flox/flox*^ mice containing eArchT3.0 in LH^*Pdx1*^ neurons and optic fiber implants over PVH underwent two 10-min trials: for Mock Inhibition trials, mice were fasted for 24 h and re-fed under ‘mock inhibition’ (optic fiber cable attached to head but no light delivered) for 10 min. For +Light trials, mice were fasted for 24 h and re-fed during alternating, consecutive light-off (1 min) followed by light-on (556 nm, ~10 mW/mm^2^; constant-on for 1 min) periods for 10 min. Trials were done at least a week apart. Mock inhibition trial was repeated a second time to verify randomness of time spent eating in each time period. Videos were recorded during trials and later manually quantified for time spent feeding and grooming by an observer.

*Pdx1-Cre::Vgat* ^*flox/flox*^ mice containing eArchT3.0 in LH^*Pdx1*^ neurons and optic fiber implants over PVH performed 4 trials (performed on separate days, in randomized order): Trial A, Using a spray bottle filled with sterile water, mice were water sprayed with two squirts directed to the face, belly, and back (two squirts per area) to induce grooming^9^. They were then placed in a bare cage with a single food pellet and observed for 10 min, with laser cable attached to the head delivering continuous 447 nm light pulses (10 s light-on, followed by 2 s light-off, 5–15 mW/mm^2^); Trial B, same as trial A, except 556 nm light pulses were delivered instead (10 s light-on, followed by 2 s light-off, 5–15 mW/mm^2^); Trials C and D, either 447 nm or 556 nm light was delivered during testing as in trials above; however, although mice were handled similarly to water spray experiments before testing, they did not receive any water sprays during these trials. Mice were acclimated to the bare cage with optic fiber cables attached to the head for 5 min prior to water spray or handling. Videos were recorded for each trial and later analyzed for grooming and feeding behaviors.

*Sim1-Cre* mice injected with AAV-FLEX-EGFP or AAV-FLEX-iC++ -EYFP virus in the PVH were used in feeding and stress-induced grooming assays. For food intake assays, mice were first acclimated to a bare testing cage with optic fiber cables attached to the head for 10 min. Following acclimation, a pellet of food was added to the cage, and 473 nm light was pulsed continuously (2 s light-on, 1 s light-off; 10–15 mW/mm^2^) for 30 min. Food weight was measured before and after 30 min tests. For baseline grooming tests (No Stress+Mock Inhibition trials), mice were placed in a bare testing cage with optic fiber cables attached to head, and observed for 15 min. For stress-induced grooming assays, mice were immobilized on a cutting board with autoclave tape for 10 min to induce anxiety and stress. They were then placed in a bare testing cage with optic fiber cables attached to the head and observed for 15 min with light pulsed continuously (2 s light-on, 1 s light-off; 10–15 mW/mm^2^). Videos were taken to later quantify time spent grooming. For food intake tests, baseline grooming, and stress-induced grooming experiments, GFP and iC++ mice were tested side-by-side and in pairs when possible.

### Feeding vs. grooming competition experiments

To study whether photostimulation of glutamatergic LH^*Pdx1-ChR2*^→PVH fibers affects fast- refeeding, *Pdx1-Cre::Vgat* ^*flox/flox*^ mice receiving ChR2 injections in LH^*Pdx1*^ neurons and optic fiber implants over PVH were placed in a bare testing cage with a pellet of food ~1–3 h before the beginning of the dark cycle. The same mice underwent 3 separate trials, lasting 30 min each. Food intake was measured following each 30-min trial and videos were recorded for grooming behavior analysis. For one of the trials, food intake was measured for ad libitum-fed mice following 30 min of mock stimulation (Trial A). In Trial B, mice were fasted for 24 h and re-fed under mock stimulation. For Trial C, mice were fasted for 24 h and then re-fed under light-on conditions (473 nm, ~5–10 mW/mm^2^ light: 5 Hz, 100 ms) for the final trial. The order of trials A-C was not the same for every mouse (to ensure a level of randomization), and mice were never fasted more than once per week. During mock stimulation trials, optic fiber cables were attached to the heads of the mice but no light was delivered. Trial A and Trial B or C occurred at least 24 h apart, and Trials B and C occurred at least a week apart. An observer blind to the experimental conditions manually quantified the time spent grooming during each 30 min trial.

To study the effects of photostimulating GABAergic LH^*Pdx1-ChR2*^→PVH fibers during self-grooming, *Pdx1-Cre::Vglut2* ^*flox/flox*^ mice containing ChR2 in LH^*Pdx1*^ neurons and optic fiber implants over PVH underwent three 11-min trials, consisting of alternating consecutive 1 min light-off followed by 1 min light-on (473 nm, ~5–10 mW/mm^2^ light: 5 Hz, 100 ms) periods or mock stimulation (optic fiber cable attached to head but no light delivered). Prior to the trials, mice received either no water sprays or sterile water sprays with a spray bottle directed to the face, belly, and back (2 squirts per area) to induce grooming. During the first trial, mice received water sprays and then were tested with mock stimulation. For the second trial, mice received water sprays prior to testing with light-off/light-on. For the third trial, no water spray was delivered and mice were tested with light-off/light-on. During trials, videos were recorded and time spent grooming and feeding was later quantified by an observer blind to the experimental conditions. Trials were performed on separate days. To test if photostimulation of PVH^*Sim1-ChR2*^ neurons interferes with fast-refeeding, *Sim1-Cre* and *Sim1-Cre::Vglut2* ^*flox/flox*^ containing ChR2 in PVH neurons and optic fibers over PVH were fasted for 24 h and re-fed during alternating consecutive light-off (1 min) followed by light-on (473 nm, ~5–10 mW/mm^2^ light: 5 Hz, 10 ms) periods for 11 min. Videos of the trials were recorded and time spent grooming and feeding was later quantified by an observer blind to genotype.

### In situ hybridization (ISH)

Manual RNAscope® assay (Advanced Cell Diagnostics, INC., Newark, CA, USA) was used to visualize *Vglut2* and *Vgat* transcript in fresh frozen brain slices. The digoxigenin-labeled cRNA probes were generated against mouse *Vgat* mRNA and *Vglut2* mRNA sequence covering exon 2, which is flanked by two *loxp* sites. ISH was performed as we previously described for validating the deletion of *Vglut2* mRNA in Sim1 neurons^30,56^. Briefly, fresh brain was harvested from *Vglut2* ^*flox/flox*^ and *Pdx1-Cre::Vglut2* ^*flox/flox*^ animals and frozen on dry ice prior to embedding in cryo-embedding medium (OCT). Brains in OCT were immediately frozen over dry ice. Embedded tissue was then equilibrated at −20 °C for 30 min–1h prior to sectioning on a cryostat. 15 µm-thick fresh frozen sections were cut with the cryostat and mounted onto slides. The slides were then immersed in chilled 10% buffered formalin for 15 min at 4 °C. Thereafter, the sections were dehydrated by immersing in 50% EtOH, 70% EtOH, and 100% EtOH for 5 min each at room temperature. After allowing slides to dry, a hydrophobic barrier was drawn around tissue with a hydrophobic barrier pen. The slides were then placed on a hybridization humidifying rack and treated with protease pretreatment solution for 30 min at room temperature. After pretreatment, slides were washed twice with fresh 1X PBS in a slide rack. PBS was gently tapped away from slides prior to applying the hybridization probe for *Vglut2*. The slides were placed in the humidifying rack and allowed to incubate for 2 h at 40 °C in a hybridization incubator. After hybridization, slides went through a series of washes with 1X RNAscope® wash buffer, followed by 4 amplification steps of the hybridization signal. After the wash and amplification step, slides were counterstained with DAPI and cover-slipped with Prolong Gold Anti-fade mounting medium (Life Technologies). *Vglut2* signal in matched sections of control and knockout groups was visualized with confocal microscopy (Leica TCS SP5; Leica Microsystems, Wetzlar, Germany).

### Brain tissue preparation, imaging, and post-hoc analysis

After behavioral experiments were completed, study subjects were anesthetized with a ketamine/xylazine cocktail (100 mg/kg and 10 mg/kg, respectively) and subjected to transcardial perfusion. During perfusion, animals were flushed with 20 mL of saline prior to fixation with 20 mL of 10% buffered formalin. Freshly fixed brains were then extracted and placed in 10% buffered formalin in 4 °C overnight for post-fixation. The next day, brains were transferred to 30% sucrose solution and allowed to rock at room temperature for 24 h prior to sectioning. Brains were frozen and sectioned into 30 µm slices with a sliding microtome and mounted onto slides for post-hoc visualization of injection sites and cannula placements. Injection sites were marked on an atlas where –EYFP or –EGFP fusion products were the densest. The location of cannula implants were noted by prominent lesion sites that extended over the rostro-caudal axis of the PVH. Mice with missed injections to the LH or misplaced optic fibers over the PVH were excluded from behavioral analysis. Representative pictures of LH and PVH injection sites and cannula placements were visualized with confocal microscopy (Leica TCS SP5; Leica Microsystems, Wetzlar, Germany). Microinjection delivery to PVH was confirmed by injecting blue ink through the fluid injection cannula prior to perfusion. Blue ink perfusion in the PVH area was confirmed with a bright field microscope (Zeiss Axio Scope with Axiocam 506 color camera; Carl Zeiss Microscopy, Jena, Germany).

### Statistics

GraphPad Prism 7.00 (GraphPad Software, Inc., La Jolla, CA, USA) was used for all statistical analyses and construction of graphs. Two-way repeated measures or regular two-way ANOVA followed by Dunnett’s or Sidak’s multiple comparisons tests were used for group comparisons. Single variable comparisons were made by paired or unpaired two-tailed Student’s *t*-tests, ratio paired Students's *t*-tests, Mann Whitney tests, or one-way ANOVA followed by Dunnett’s or Tukey’s multiple comparison post-hoc tests. Error bars in graphs were represented as mean ± s.e.m. Sample size was chosen based on previously published work. All tests met assumptions for normal distribution, with similar variance between groups that were statistically compared. N values represent final number of animals used in experiments following genotype verification and post-hoc brain validation of injection sites/cannula placements.

### Data availability

All relevant and supporting data are freely available upon reasonable request from the corresponding author.

## Electronic supplementary material

Supplementary Information

Description of Additional Supplementary Information

Supplementary Movie 1

Supplementary Movie 2

Supplementary Movie 3

Supplementary Movie 4

Supplementary Movie 5

Supplementary Movie 6
