# Supplementary material for: A neural basis for antagonistic control of feeding and compulsive behaviors
Source: Nat Commun. 2018 Jan 4;9:52. doi: 10.1038/s41467-017-02534-9 (PMC5754347; doi:10.1038/s41467-017-02534-9)
Supplement: Supplementary file 1 — Supplementary Information [file 41467_2017_2534_MOESM1_ESM.pdf]

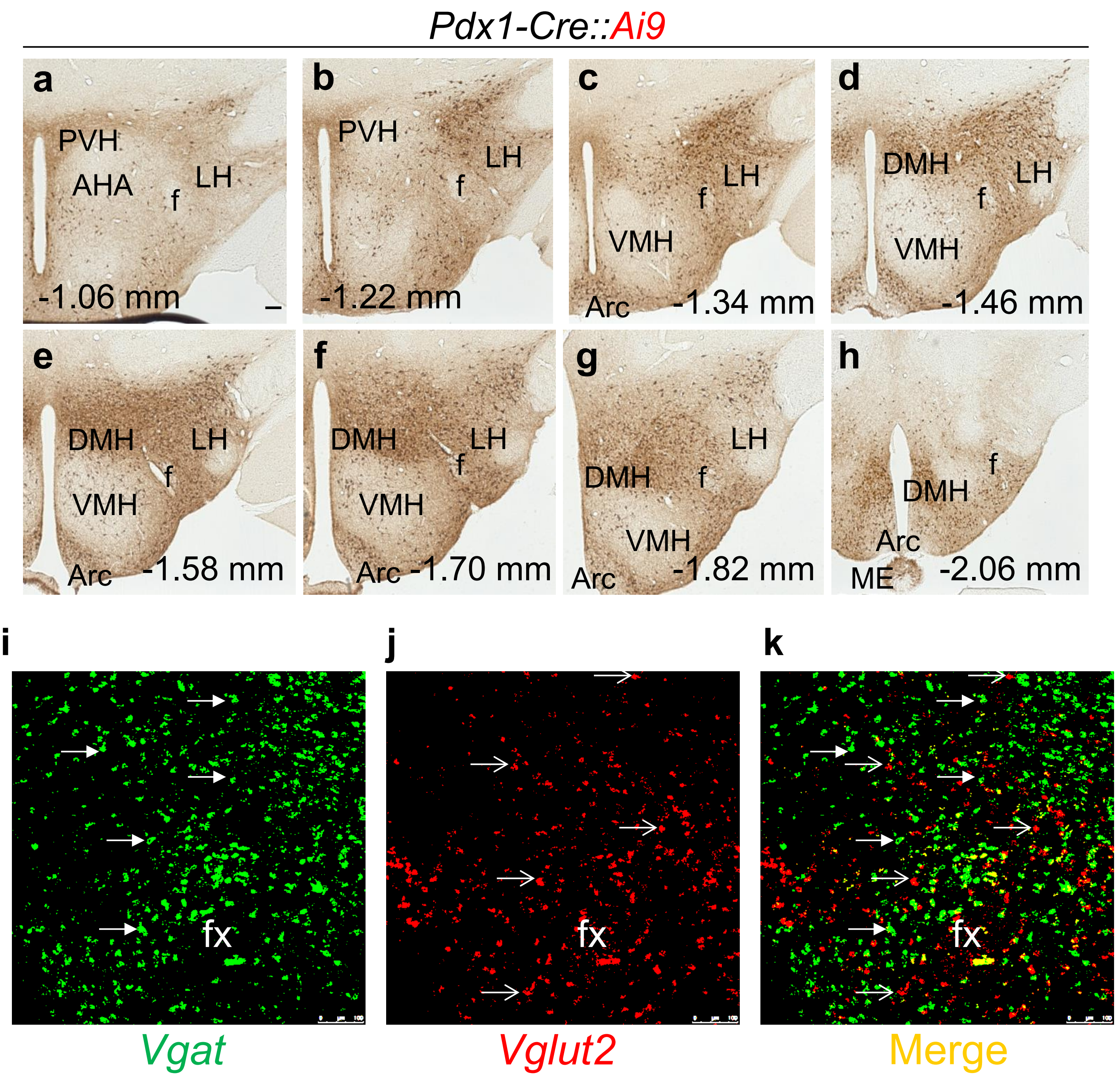

Supplementary Figure 1 *Pdx1-Cre* expression in hypothalamic brain regions, including LH, which contain segregated GABA and glutamatergic neuron populations. *Pdx1-Cre* mice were bred to Gt(ROSA)26Sortm9(CAG-tdTomato)Hze, also known as Ai9 reporter mice, to allow red-fluorescent protein (RFP) visualization in cre-positive neurons and fibers. In a rostral to caudal fashion, (**a-h**) show RFP expression in hypothalamic neurons, including those in the LH, and projection fibers located in the PVH. Prominent *Pdx1-Cre* expression in LH is noted in the medial to caudal portions of the LH (-1.22 to -1.82 mm Bregma, **b-g**), but is not seen in LH area anterior to Bregma -1.22 mm. Other prominent *Pdx1-Cre* positive neurons are seen in the DMH and Arc. Measurements indicate millimeter anterior-posterior distance relative to Bregma. Arc, arcuate nucleus; DMH, dorsomedial hypothalamus; f, fornix; LH, lateral hypothalamus; VMH, ventromedial hypothalamus. (**i-k**) Double in situ hybridization of *Vgat* (**i**) and *Vglut2* (**j**) in the LH area of a wild-type mouse. Merged image (**k**) shows most *Vgat* and *Vglut2* expressing neurons in LH do not overlap. fx, fornix

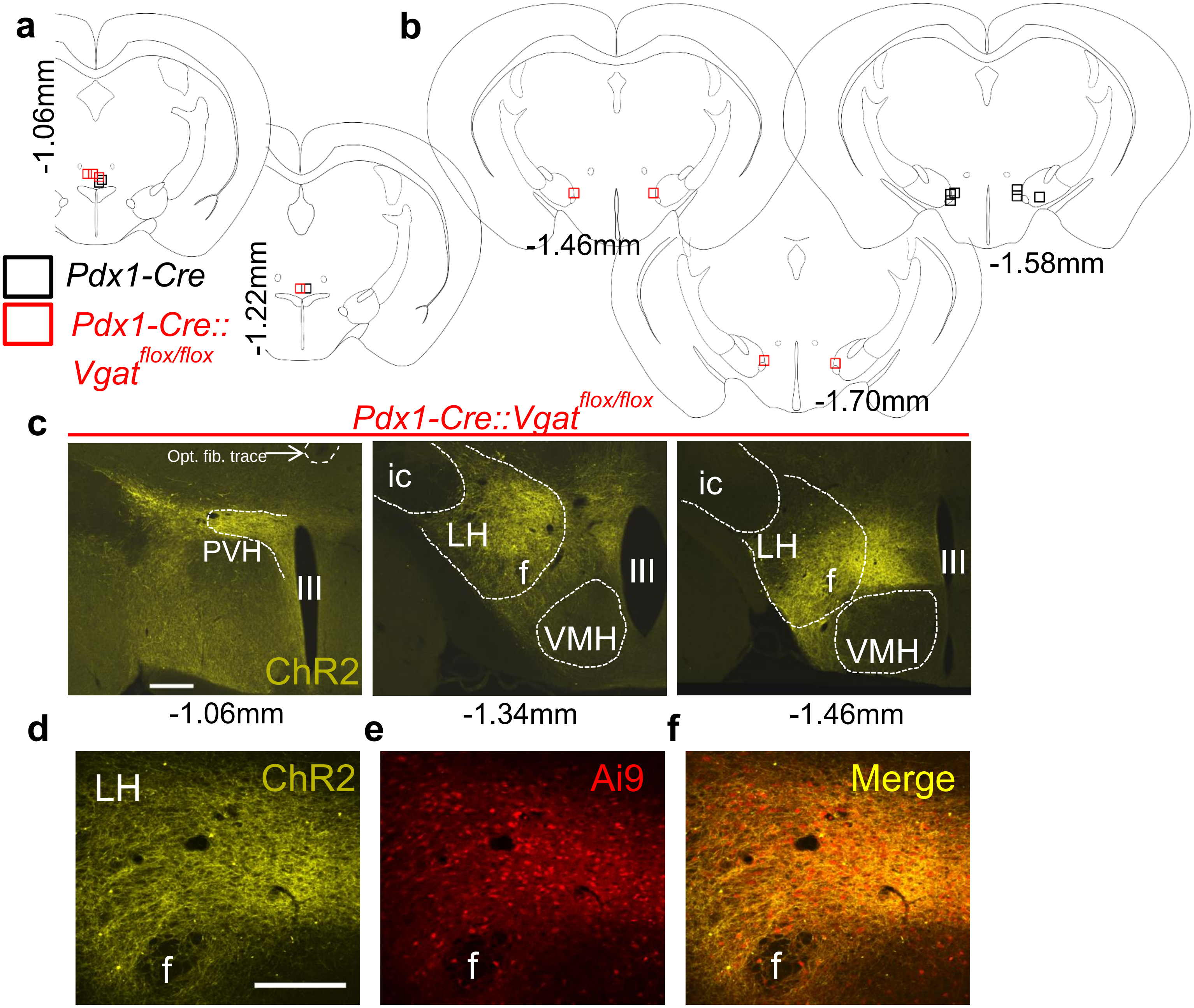

Supplementary Figure 2 ChR2 expression in LH^{Pdx1} neurons. (**a**) Post-hoc analysis in coronal brain sections revealed optic fiber traces over caudal portions of the PVH in *Pdx1-Cre* mice (black outlined boxes) and *Pdx1-Cre::Vgat*$^{flox/flox}$ mice (red outlined boxes) used for experiments in **Figure 2**. (**b**) Shows approximate ChR2 injection sites, as determined by dense eYFP expression, in the same mice. (**c**) Representative images taken from brain slices of a *Pdx1-Cre::Vgat*$^{flox/flox}$ mouse used in experiments for **Fig. 2** reveal ChR2 expression in PVH projection fibers and optical fiber implantation above PVH (opt. fib. trace, arrow) (left image) and ChR2 expression in LH^{Pdx1} neurons (middle and right images). (**a-c**): millimeter measurements indicate anterior-posterior distance relative to Bregma. III, third ventricle; f, fornix; ic, internal capsule; LH, lateral hypothalamus; PVH, paraventricular hypothalamus; VMH, ventromedial hypothalamus. Scale bar = 300µm. (**d-f**) Representative coronal brain slice images from a *Pdx1-Cre::Ai9* reporter mouse used in **Fig. 2** experiments show ChR2 (**d**) and Ai9 (**e**) expression overlapping in the LH region (**f**). Scale bar = 250 µm.

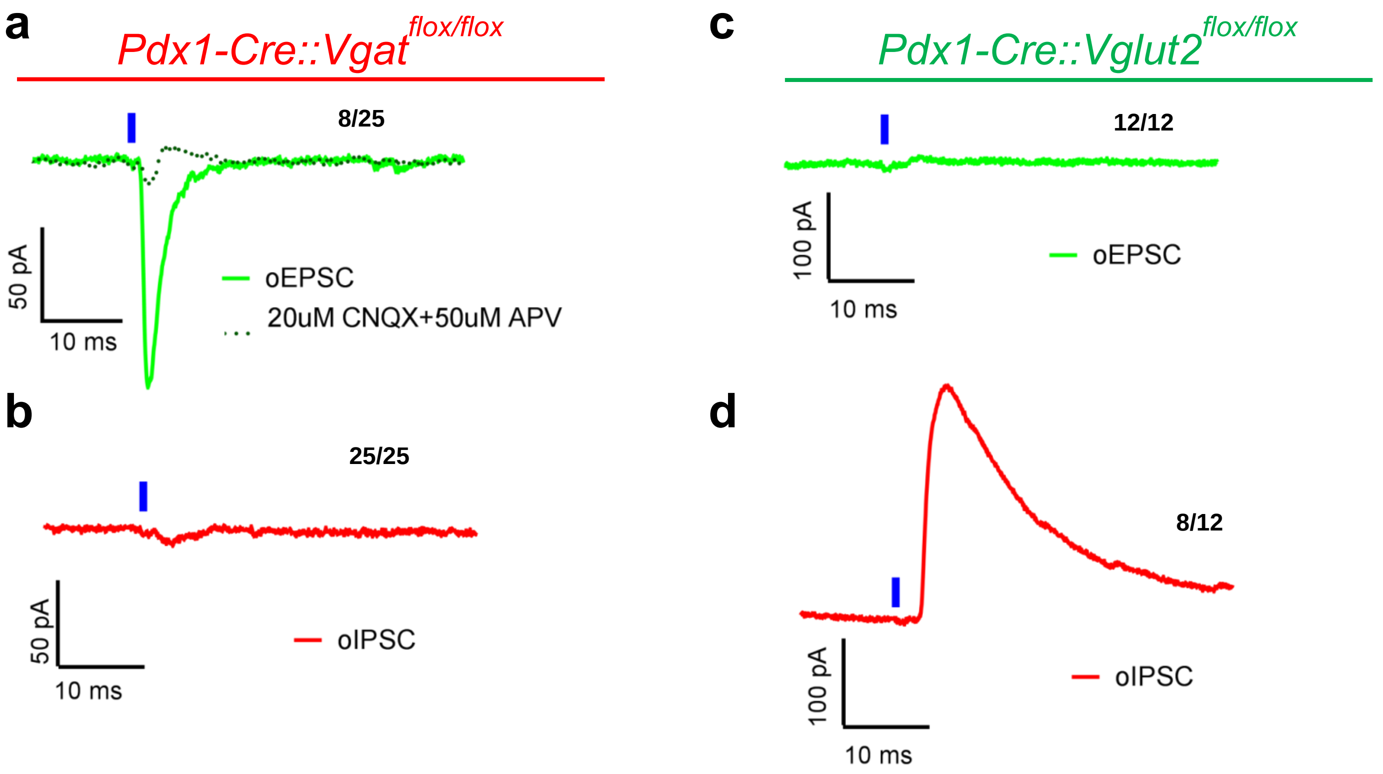

Supplementary Figure 3 Inhibitory and excitatory post-synaptic currents in PVH elicited by blue light stimulation of LH$^{Pdx1\text{-}ChR2}$ fibers require vesicular GABA transporter (Vgat) and vesicular glutamate transporter 2 (Vglut2), respectively. (**a**) Voltage clamp recordings in PVH brain slices of *Pdx1-Cre::Vgat*$^{flox/flox}$ mice reveal optically-evoked excitatory post-synaptic currents (oEPSCs, which can be blocked by ionotropic glutamate receptor blockers CNQX+APV), but not optically-evoked inhibitory post-synaptic currents (oIPSCs) (**b**). Conversely, PVH recordings in *Pdx1-Cre::Vglut2*$^{flox/flox}$ brain slices show light can elicit IPSCs (**d**) but not EPSCs (**c**). Blue ticks indicate 1-ms blue light pulse.

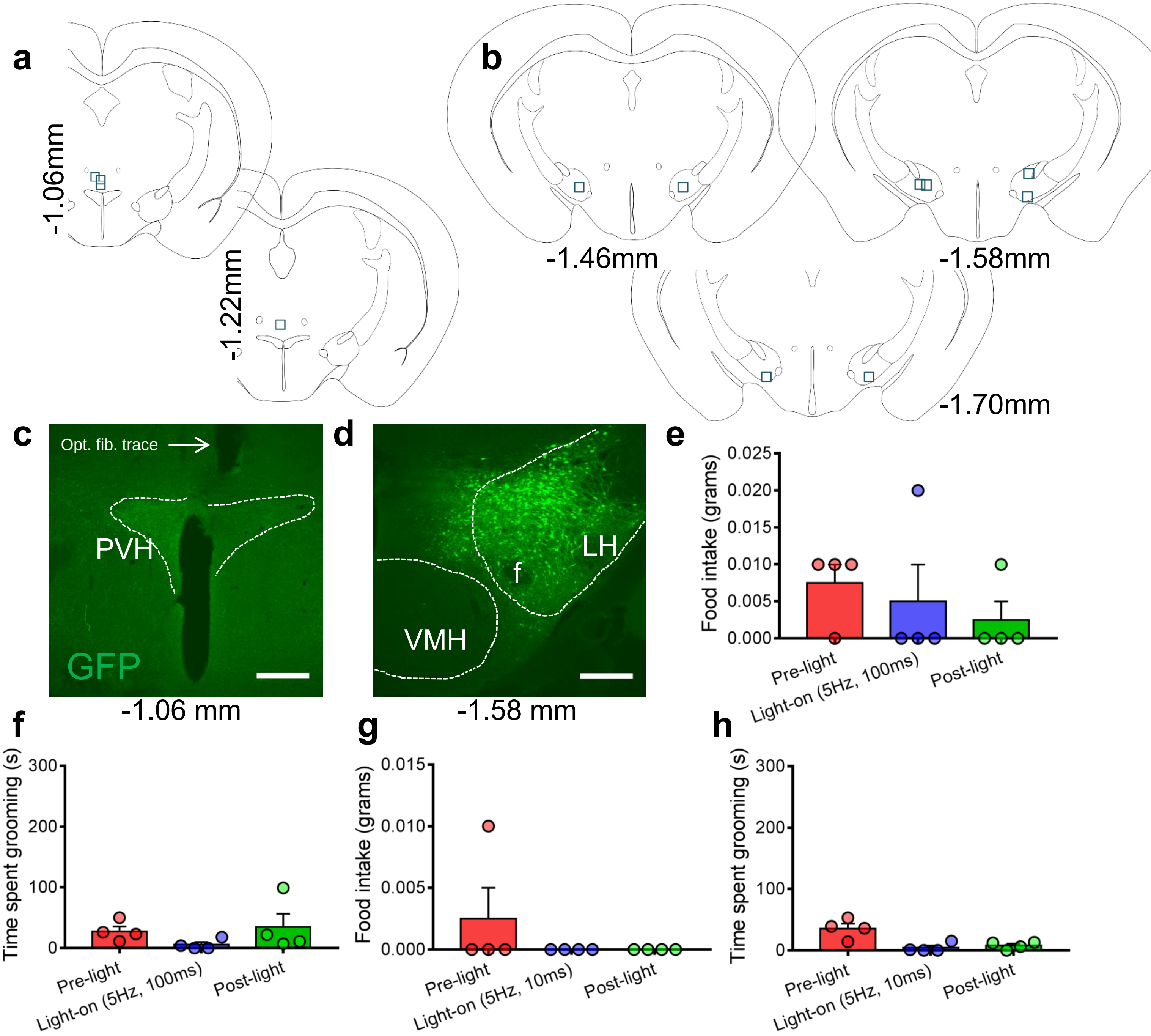

Supplementary Figure 4 Photostimulation of LH$^{Pdx1\text{-}GFP}$ expressing fibers in PVH does not increase feeding or grooming behaviors. (**a**) Post-hoc analysis of brain slices show optic fiber placements (blue outlined boxes) above caudal PVH regions in *Pdx1-Cre* mice injected with cre-dependent GFP virus in LH. (**b**) Shows approximate injection locations of GFP (blue outlined boxes) in the same mice. Representative images from a *Pdx1-Cre*::GFP mice (n=4) showing GFP-expressing fibers in PVH and optical fiber implantation above PVH (opt. fib. trace, arrow) (**c**) and injection site in LH (**d**). Scale bar = 300µm. *In vivo* photostimulation of LH$^{Pdx1\text{-}GFP}$→PVH fibers does not cause significant increases in either feeding (**e, g**) or grooming behaviors (**f, h**), irrespective of light stimulation protocol (5Hz, 10 vs. 100 ms). Repeated measures ANOVA: (**e**) Light epoch $F(2, 6) = 0.4286$, $P=0.6699$; (**f**) Light epoch $F(2, 6) = 1.644$, $P=0.2696$; (**g**) Light epoch $F(2, 6) = 1$, $P=0.4219$ (**h**) Light epoch $F(2, 6) = 7.057$, $P=0.0265$). Data presented as ±s.e.m.

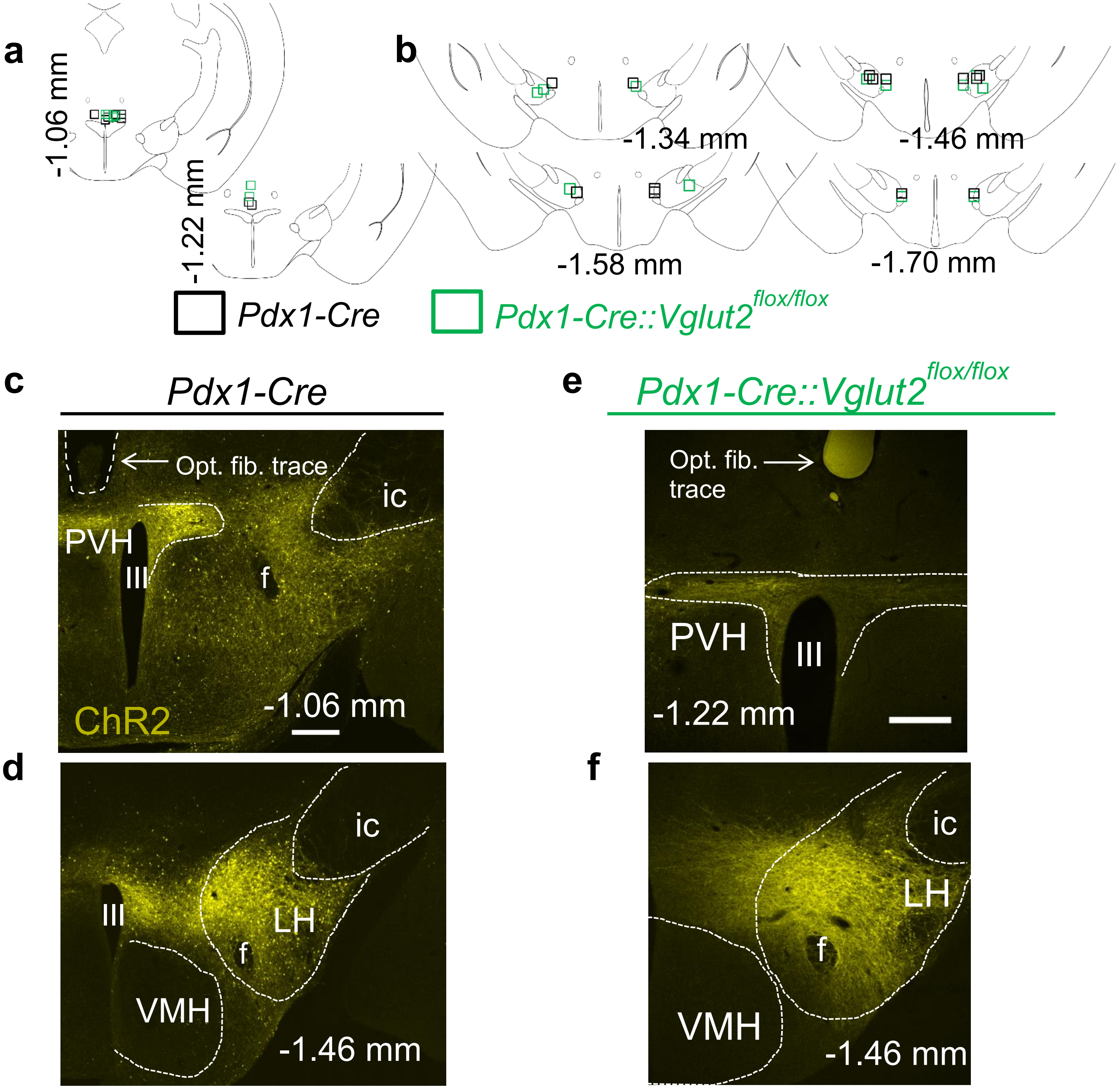

Supplementary Figure 5 ChR2 expression in LH neurons and PVH projection fibers in *Pdx1-Cre* and *Pdx1-Cre::Vglut2^flox/flox^* mice. (**a**) Post-hoc analysis in brains of mice used for **Figure 3** experiments shows optic fiber placements above caudal PVH region (indicated by black and green outlined boxes). (**b**) The same mice received ChR2 injections consistently targeted in the LH region (black and green outlined boxes). Representative PVH brain slice image of a *Pdx1-Cre* mouse showing ChR2-expressing fibers in rostral PVH and optical fiber implantation above PVH (opt. fib. trace, arrow) (**c**) and ChR2 injection site in LH (**d**). Representative brain slice images from a *Pdx1-Cre::Vglut2^flox/flox^* mouse reveals ChR2 fibers in caudal PVH and optical fiber implantation above PVH (left image) (**e**) and ChR2 injection site in LH (**f**). Scale bar = 300µm. III, third ventricle; f, fornix; ic, internal capsule; LH, lateral hypothalamus; PVH, paraventricular hypothalamus; VMH, ventromedial hypothalamus.

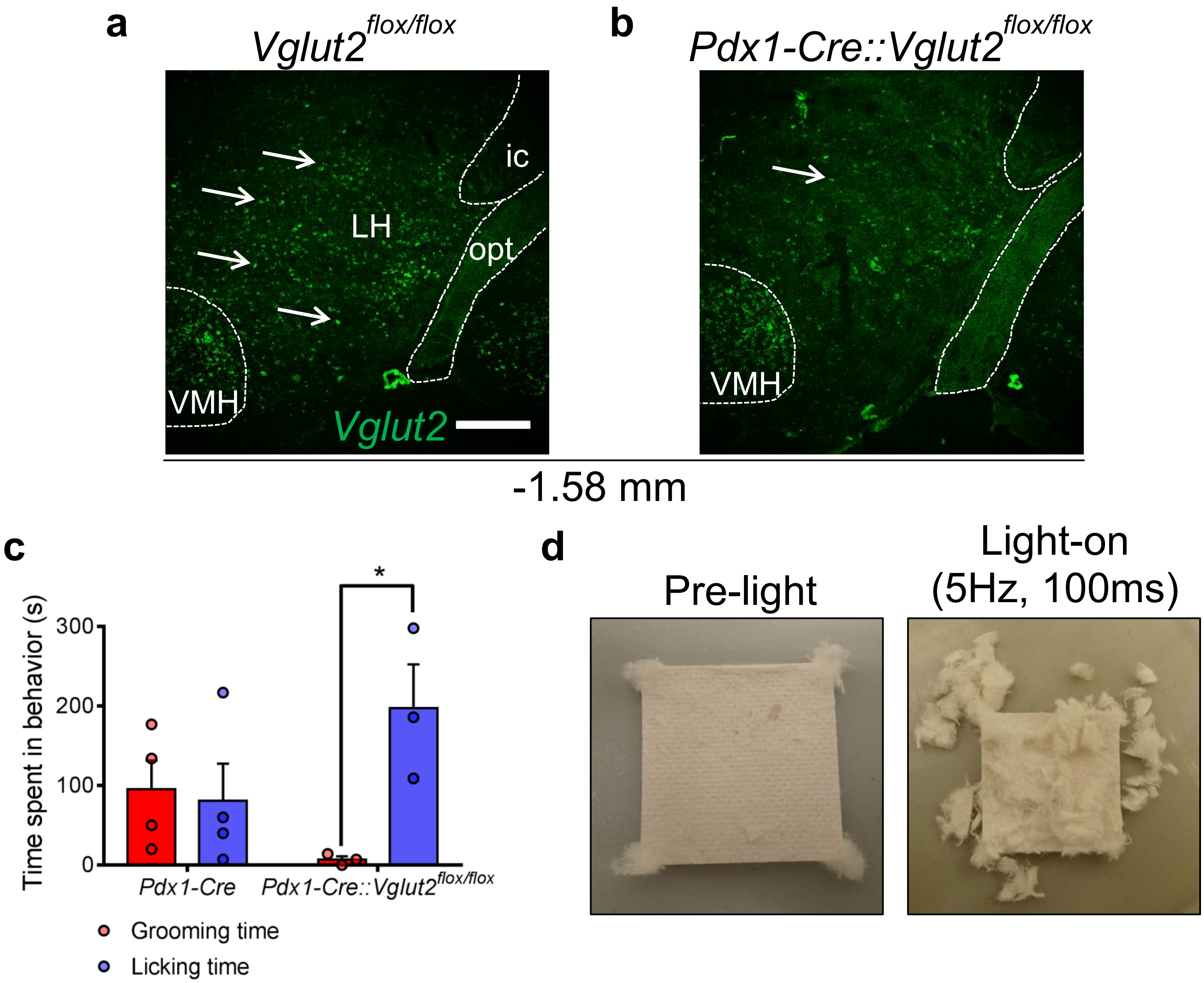

Supplementary Figure 6 Photostimulation of GABAergic LH→PVH projections causes chewing and licking behavior. (**a-b**) *In situ* hybridization for *Vglut2* mRNA in fresh frozen coronal brain slices shows numerous neurons with *in situ* fluorescent signal (arrows) in the LH region of $Vglut2^{flox/flox}$ mice (**a**). In contrast, in $Pdx1\text{-}Cre::Vglut2^{flox/flox}$ mice, the number of neurons with *in situ* fluorescent signal in a matching LH-containing section is dramatically reduced while it is not changed in neighboring VMH area (**b**). Scale bar = 300µm. Millimeter measurement indicates anterior-posterior distance from Bregma. ic, internal capsule; LH, lateral hypothalamus; opt, optic tract; VMH, ventromedial hypothalamus. (**c**) When mice were placed in a bare cage (no food or bedding), *Pdx1-Cre* mice spent approximately equal amounts of time grooming and aimlessly licking the floors and sides of the cage upon $LH^{Pdx1\text{-}ChR2}$→PVH photostimulation (5Hz, 100ms); however, $Pdx1\text{-}Cre::Vglut2^{flox/flox}$ spent significantly more time licking the cage than grooming upon the same light stimulation (two-way ANOVA; Interaction F (1, 10) = 5.984, P=0.0345; Sidak's multiple comparisons test: Grooming time vs. Licking time ($Pdx1\text{-}Cre::Vglut2^{flox/flox}$) *p<0.05). Data presented as ± s.e.m. (**d**) A portion of the *Pdx1-Cre* and $Pdx1\text{-}Cre::Vglut2^{flox/flox}$ mice violently chewed on a square piece of bedding upon light stimulation of $LH^{Pdx1\text{-}ChR2}$→PVH circuit when food was absent. Pictures are of the same piece of bedding following 5 minutes pre-light and 5 minutes light-on stimulation.

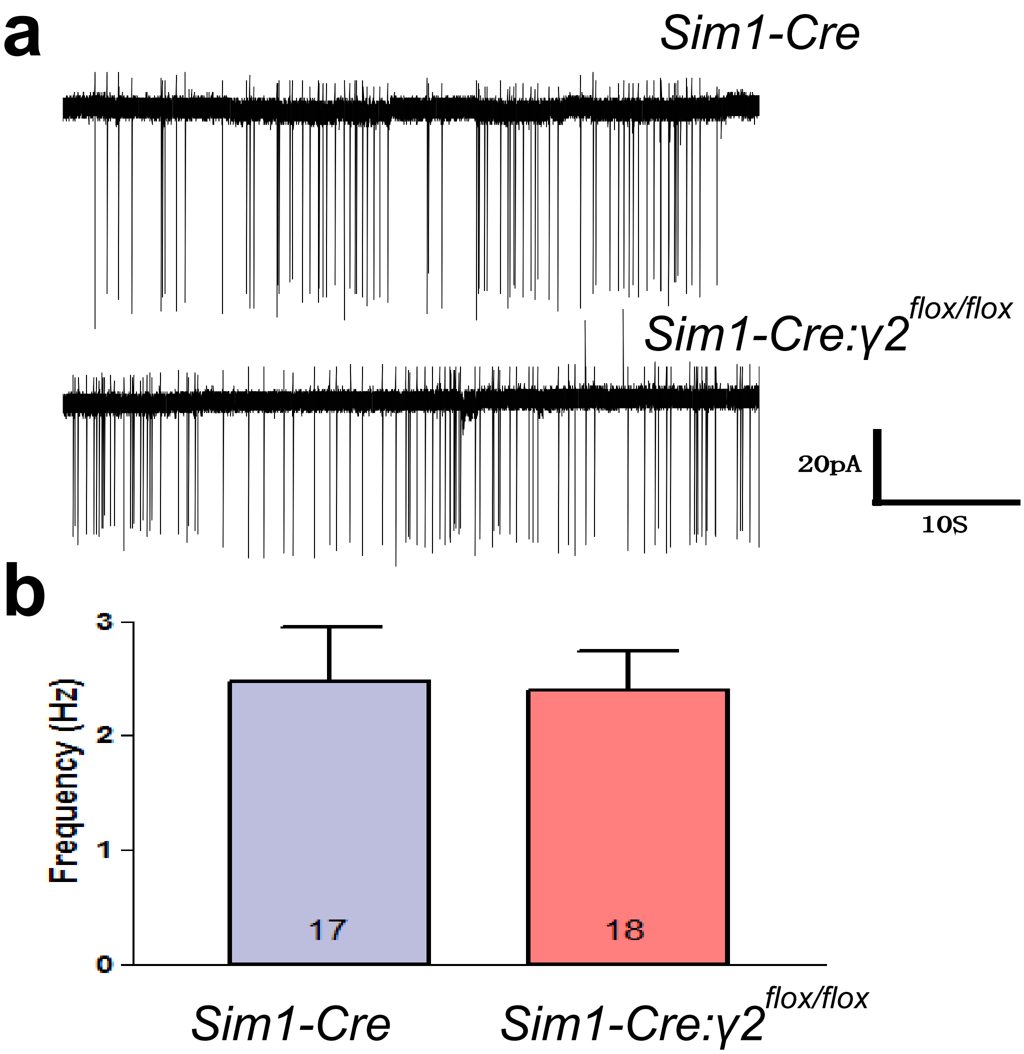

Supplementary Figure 7 Deletion of γ2 in *Sim1* neurons does not affect spontaneous firing activity in PVH^{Sim1} neurons. (**a**) Loose-patch recordings (voltage-clamp mode) of spontaneous spike discharge in PVH^{Sim1} neurons of *Sim1-Cre* (top) and *Sim1-Cre::γ2*$^{flox/flox}$ (bottom) mice. (**b**) Quantification of firing frequency in PVH^{Sim1} neurons between the indicated two groups of mice does not reveal changes in overall neuron activity between genotypes.

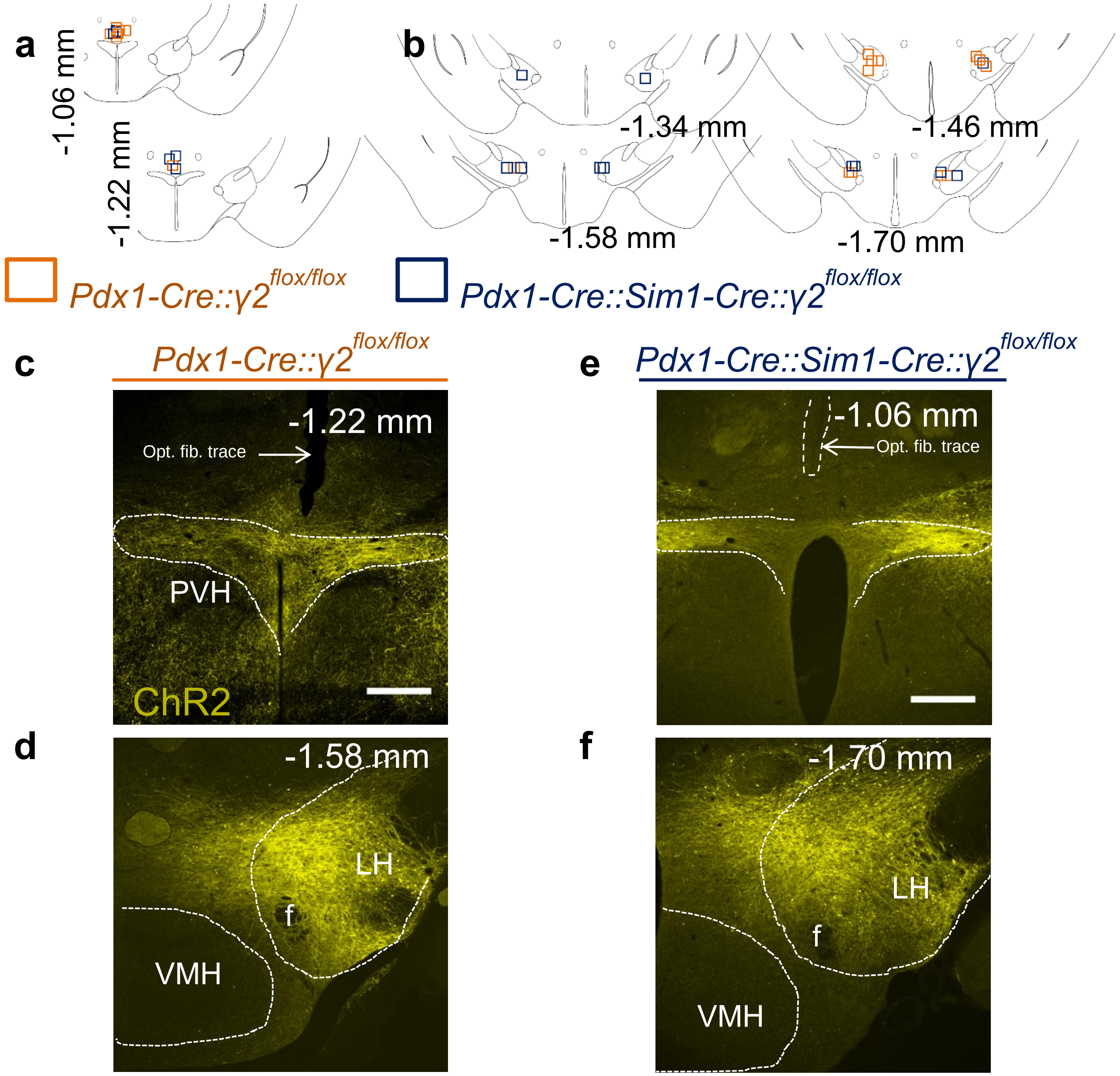

Supplementary Figure 8 ChR2 expression in LH neurons and PVH projection fibers in *Pdx1-Cre::γ2$^{flox/flox}$* and *Pdx1-Cre::Sim1-Cre::γ2$^{flox/flox}$* mice. (**a-b**) Post-hoc analysis in brains of mice used for **Figure 4d** experiments confirmed optic fiber placements above caudal PVH region (**a**) (orange and blue outlined boxes) and successful targeting of ChR2 to the LH region (**b**) (orange and blue outlined boxes). Representative coronal brain slice images from a *Pdx1-Cre::γ2$^{flox/flox}$* mouse showing ChR2 expression in caudal PVH fibers and optical fiber implantation above PVH (opt. fib. trace) (**c**) and ChR2 expression in LH injection site (**d**). Representative coronal brain slice images from a *Pdx1-Cre::Sim1-Cre::γ2$^{flox/flox}$* mouse showing ChR2-expressing fibers in caudal PVH and optical fiber implantation above PVH (opt. fib. trace, arrow) (**e**) and ChR2 expression in LH injection site (**f**). Millimeter measurements indicate anterior-posterior distance relative to Bregma. LH, lateral hypothalamus; VMH, ventromedial hypothalamus.

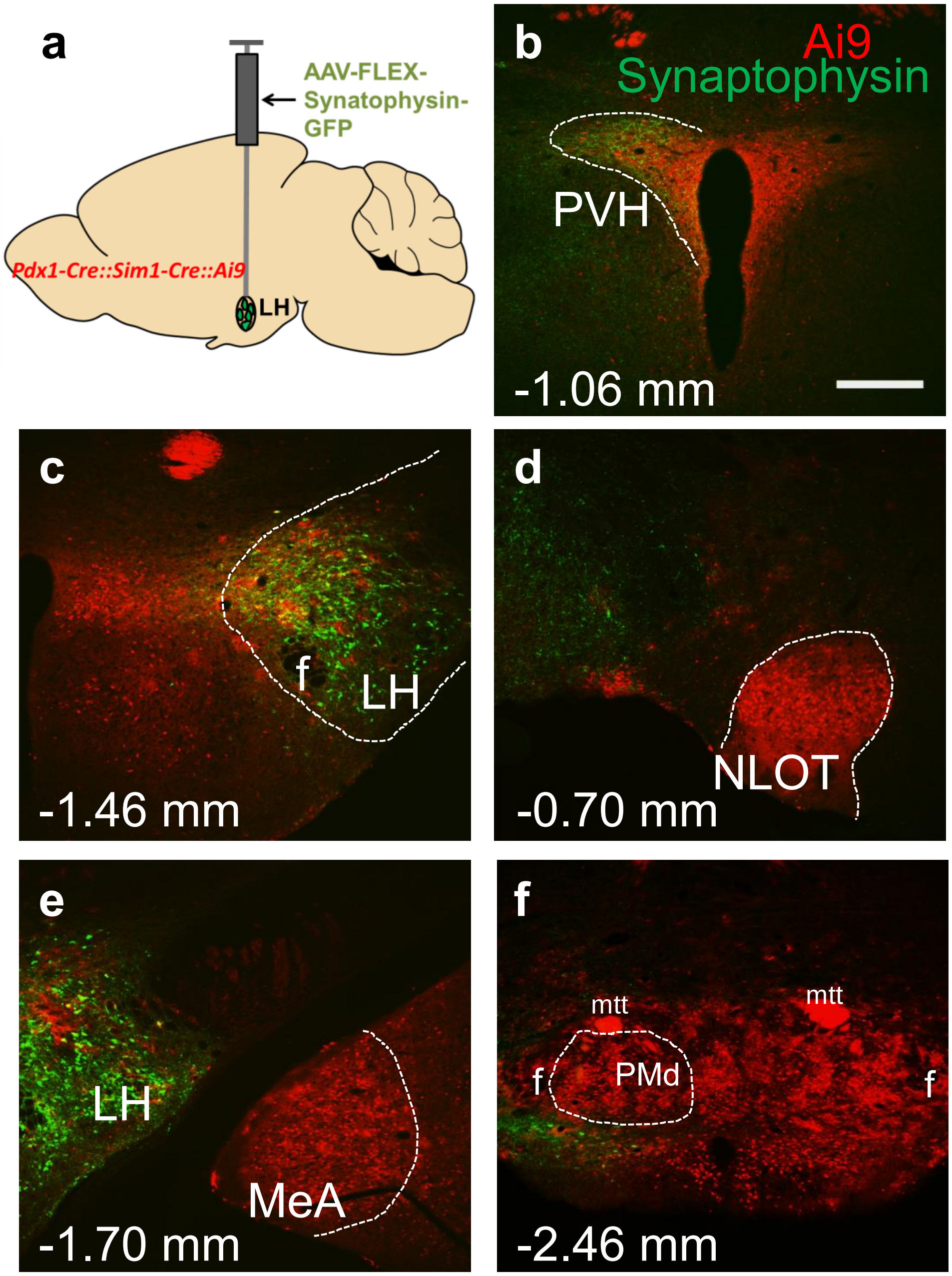

Supplementary Figure 9 LH^{Pdx1} neurons project to PVH^{Sim1} but not to other prominent *Sim1*-positive brain regions. (**a**) Schematic illustrates cre-dependent delivery of synaptophysin-GFP to LH neurons (unilateral injection) in a *Pdx1-Cre::Sim1-Cre::Ai9* mouse for anterograde tracing studies. (**b**) As expected, prominent synaptophysin-GFP contacts were made with Ai9 neurons in the PVH, suggesting LH^{Pdx1}→PVH^{Sim1} synaptic connections. (**c**) Shows successful viral delivery of synaptophysin-GFP to the LH region. (**d-f**) Prominent *Sim1*-positive sites include NLOT (**d**), MeA (**e**), and premammillary nucleus (**f**), all of which show little to no GFP overlap in the respective regions. Millimeter measurements indicate anterior-posterior distance relative to Bregma. f, fornix; LH, lateral hypothalamus; MeA, Medial Amygdala nucleus; mammillary nucleus; mtt, mammilothalmic tract; NLOT, nucleus of the lateral olfactory tract; PMd, dorsal premammillary nucleus. Scale bar = 300 µm.

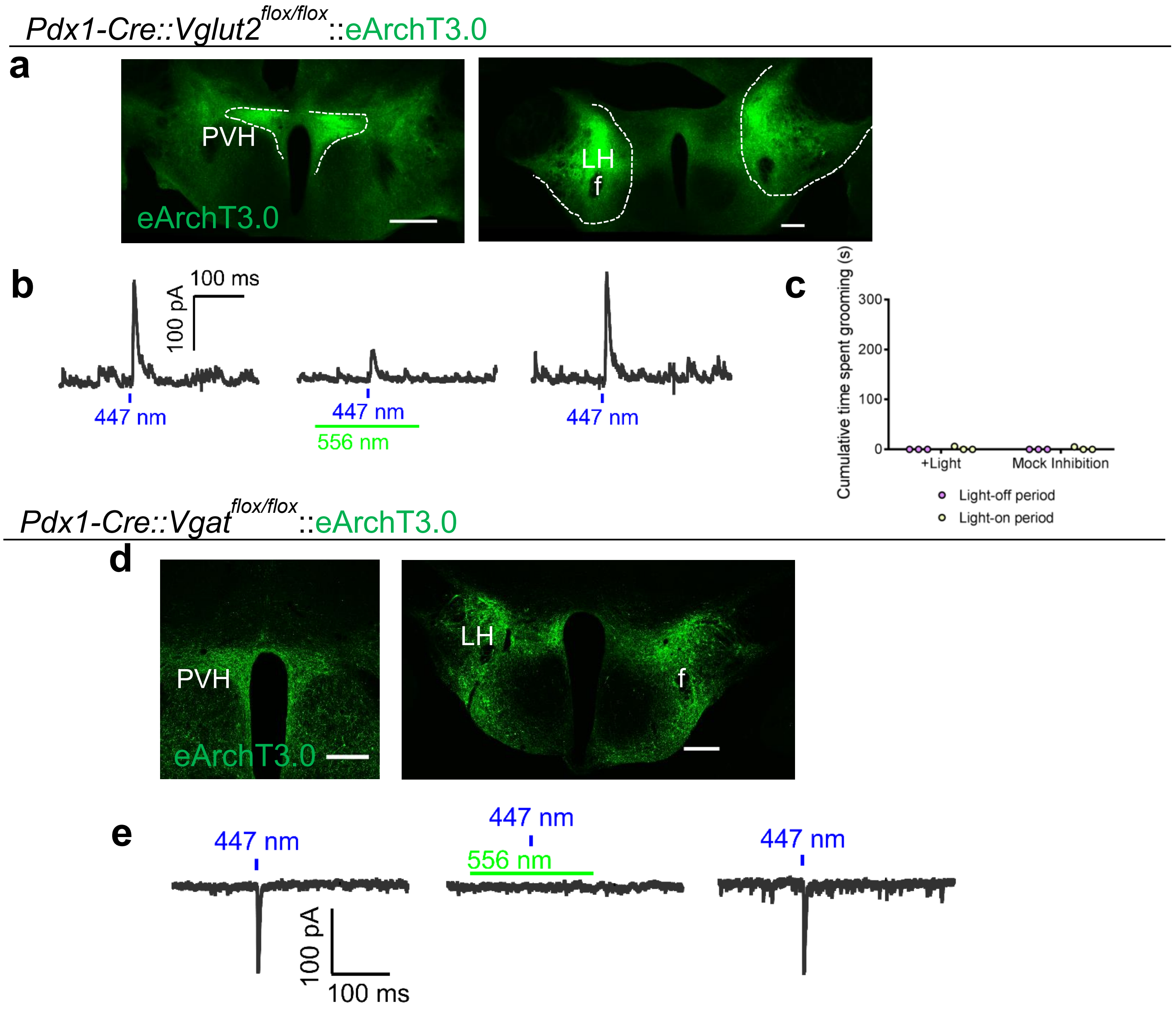

Supplementary Figure 10 eArchT3.0-mediated inhibition of GABA and glutamate release in LH^{Pdx1}→PVH terminals. (**a**) Representative images from *a Pdx1-Cre::Vglut2*$^{flox/flox}$::eArchT3.0 mouse (used in **Figure 5** for behavioral experiments) showing eArchT3.0-GFP projection fibers from LH to PVH (left image) and injection sites in LH (right). f, fornix; LH; lateral hypothalamus; PVH, paraventricular nucleus of the hypothalamus. Scale bar = 500µm. (**b**) Voltage clamp recordings in PVH brain slices of Pdx*1-Cre* mice receiving LH injections of 50:50 cre-dependent ChR2+eArchT3.0 viruses. Photostimulation-evoked IPSC with a single 2 ms pulse of blue light (left) was reversibly inhibited by illumination with 556nm light (middle, right). Traces shown were averaged responses of 5-6 sweeps. (**c**) Light (556nm) or mock inhibition of GABAergic LH^{Pdx1}→PVH terminals in *Pdx1-Cre::Vglut2*$^{flox/flox}$::eArchT3.0 mice does not significantly impact grooming time during light-off vs. light-on periods. (**d**) Representative images from *a Pdx1-Cre::Vgat*$^{flox/flox}$::eArchT3.0 mouse (used in **Figure 5** for behavioral experiments) showing eArchT3.0-GFP projection fibers from LH to PVH (left image) and injection sites in LH (right). Scale bar = 300µm. (**e**) Voltage clamp recordings in PVH brain slices of Pdx*1-Cre* mice receiving LH injections of 50:50 cre-dependent ChR2+eArchT3.0 viruses. Photostimulation-evoked EPSC with a single 1 ms pulse of blue light (left) was reversibly inhibited by illumination with 556nm light (middle, right). Traces shown were averaged responses of 5-6 sweeps.

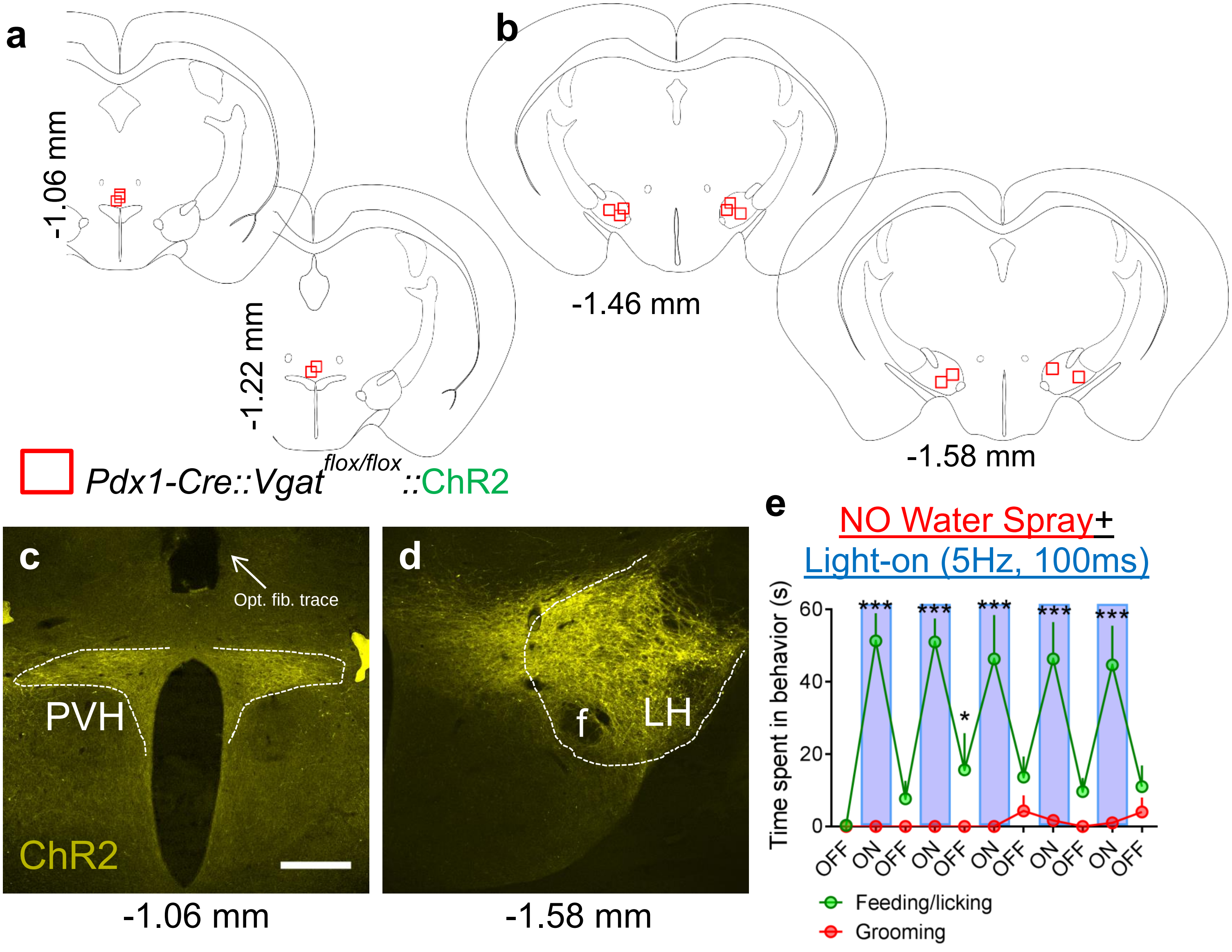

Supplementary Figure 11 ChR2 expression in LH neurons and PVH projection fibers in *Pdx1-Cre::Vgat$^{flox/flox}$* used in fast-refeeding competition experiments, and photostimulation of LH→PVH GABAergic terminals strongly promotes feeding. (**a-d**) Post-hoc analysis of optic fiber placements and LH injection sites for *Pdx1-Cre::Vgat$^{flox/flox}$*::ChR2 mice used for competition experiment in **Figure 6a-c**. Verification of optic fiber placements above PVH (**a**) and successful targeting of ChR2 to LH (**b**). Representative images show ChR2 expression in fibers projecting to PVH and optic fiber trace above PVH (**c**) and approximate injection site of ChR2 to LH region (**d**). f, fornix; LH, lateral hypothalamus; PVH, paraventricular hypothalamus. Scale bar = 300 µm. (**e**) *Pdx1-Cre::Vglut2$^{flox/flox}$*::ChR2 mice (related to **Figure 6d-f**) consistently spend most of a one-minute block feeding when laser is turned on (473nm; 5Hz, 100ms), while showing little to no increase in grooming behavior regardless of light epoch. Each Off-On epoch lasted one minute each for eleven consecutive minutes. Two-way RM ANOVA; Interaction F (10, 40) = 15.4, P<0.0001; Dunnett's multiple comparisons test: Feeding/licking (first light off vs. On-1, On-2, On-3, On-4, On-5) ***p<0.0005; Feeding/licking (first light off vs. Off-3) *p<0.05. Data presented as ±s.e.m.

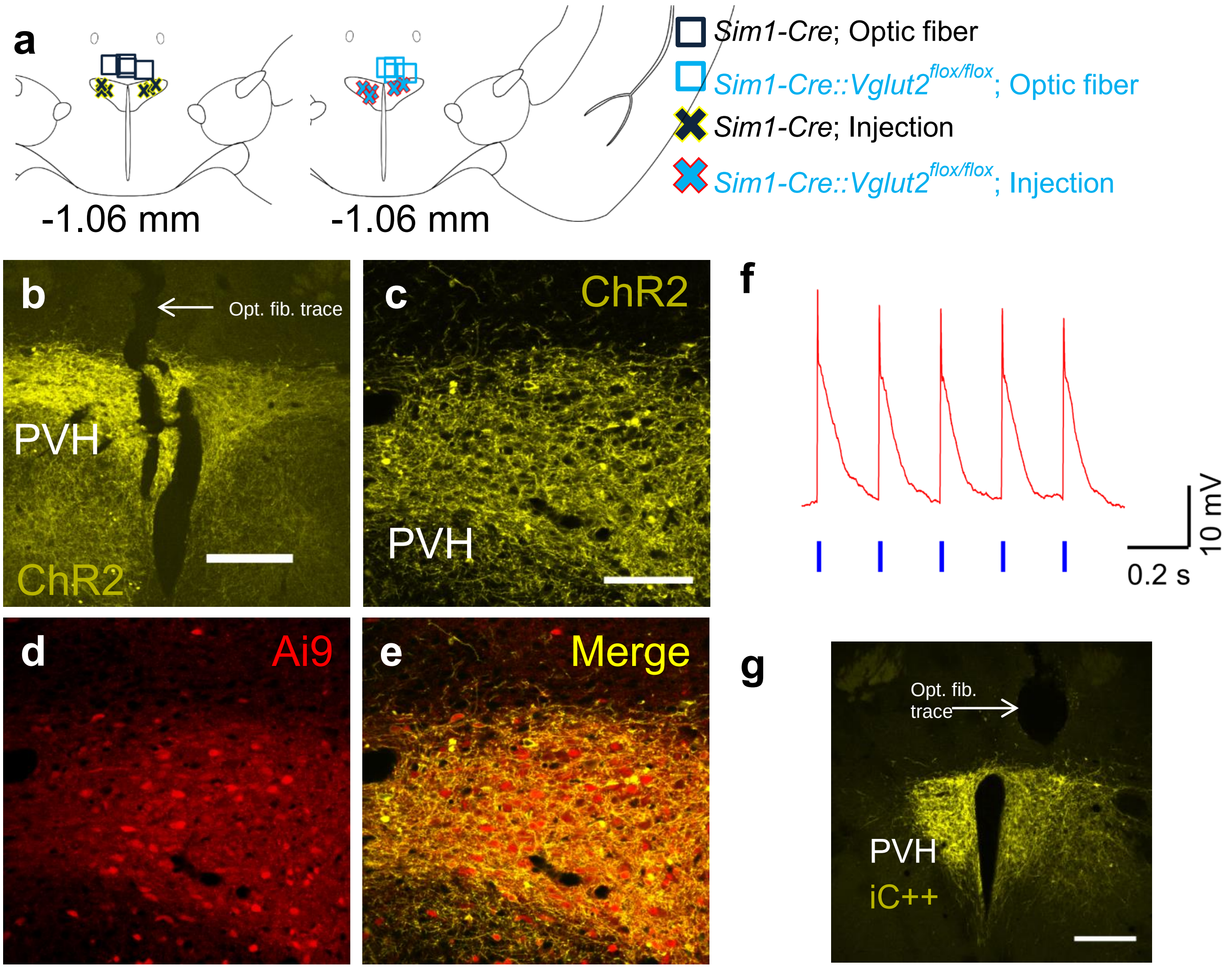

Supplementary Figure 12 ChR2 or iC++ expression in PVH^{Sim1} neurons of *Sim1-Cre* and *Sim1-Cre::Vglut2$^{flox/flox}$* mice. (**a**) Post-hoc analysis in brains of mice used in **Figure 7b-e** shows approximate injection locations of ChR2 in PVH and optical fiber implantation above PVH. Dark blue box and light blue box indicate optic fiber placements in *Sim1-Cre* and *Sim1-Cre::Vglut2$^{flox/flox}$* mice, respectively; dark blue X and light blue X indicate approximate ChR2 injection sites in *Sim1-Cre* and *Sim1-Cre::Vglut2$^{flox/flox}$* mice, respectively. (**b**) Representative image from a *Sim1-Cre::Vglut2$^{flox/flox}$* mouse showing ChR2 expression in caudal PVH and optical fiber placement above the same region. Scale bar = 300 μm. (**c-e**) Representative image from a *Sim1-Cre::Ai9* reporter mouse with ChR2 expression in PVH region. Close-up of PVH showing ChR2-eYFP fluorescence (**c**) and Ai9-RFP fluorescence (**d**). Merged image (**e**) shows ChR2 expression encircles the membranes of Ai9-positive cells. Scale bar = 100 μm. (**f**) Whole-cell current clamp traces in ChR2-positive PVH^{Sim1} cells in response to 5 Hz, 10ms blue light pulses (blue ticks = 10ms light pulse). (**g**) iC++-EYFP expression in PVH^{Sim1} neurons and optical fiber implantation above PVH. Scale bar = 300 μm.

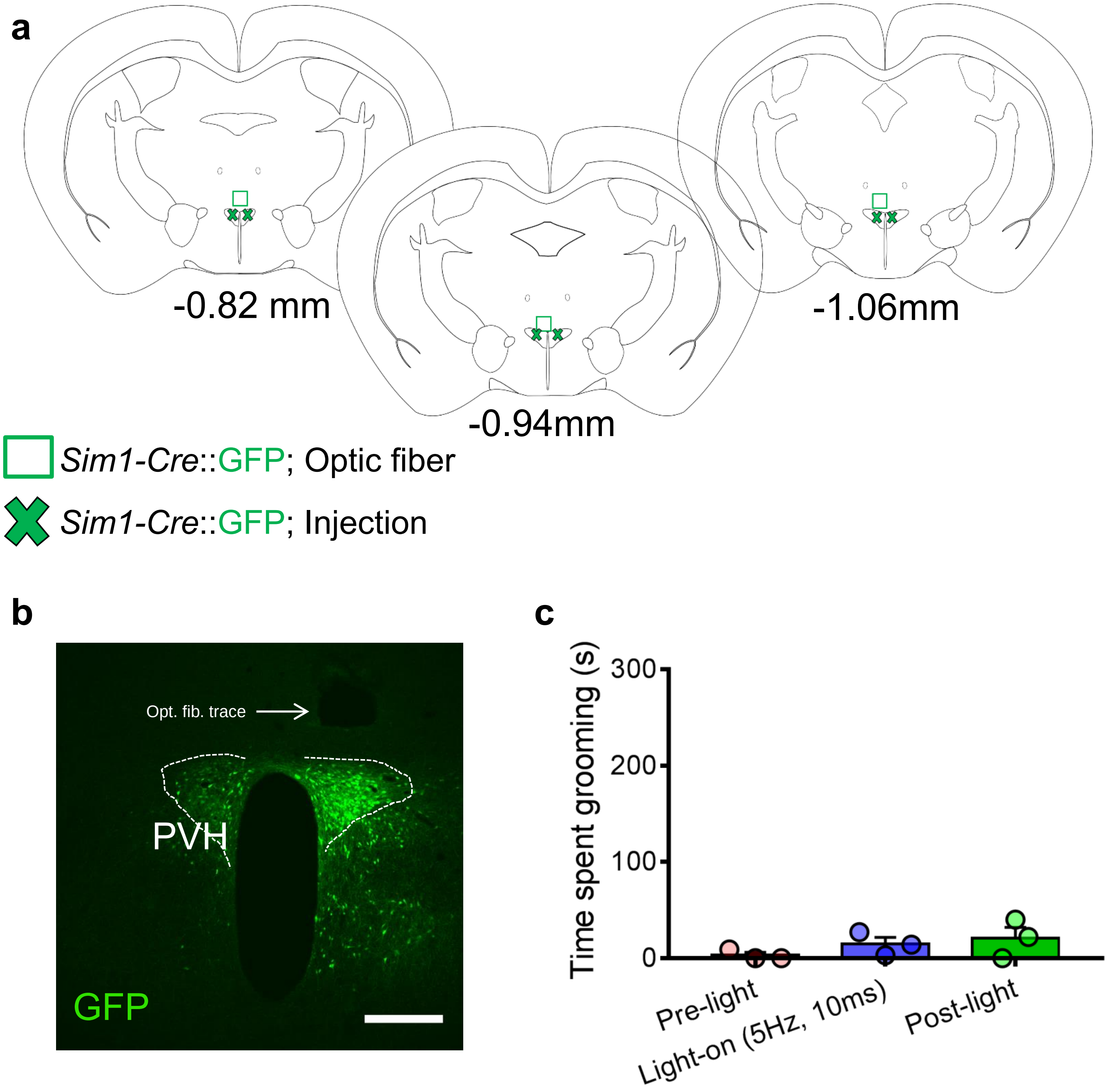

Supplementary Figure 13 Photostimulation of PVH$^{Sim1\text{-}GFP}$ neurons does not increase grooming behavior. (**a**) Shows approximate optic fiber placements (green outlined boxes) and cre-dependent GFP injection sites (green X's) in *Sim1-Cre* mice used for behavioral experiments in (**c**). (**b**) Representative image of a *Sim1-Cre* mouse showing GFP expression in PVH and optic fiber placement above the same region. Scale bar = 300 µm. (**c**) *In vivo* optical stimulation (5Hz, 10ms) of PVH$^{Sim1\text{-}GFP}$ neurons does not lead to increased grooming behavior compared to pre- and post-light epochs (repeated measures ANOVA; light epoch F (2, 4) = 2.329, P=0.2134). Each epoch lasted 5 mins for a total of 15 consecutive minutes. Data are presented as ±s.e.m.

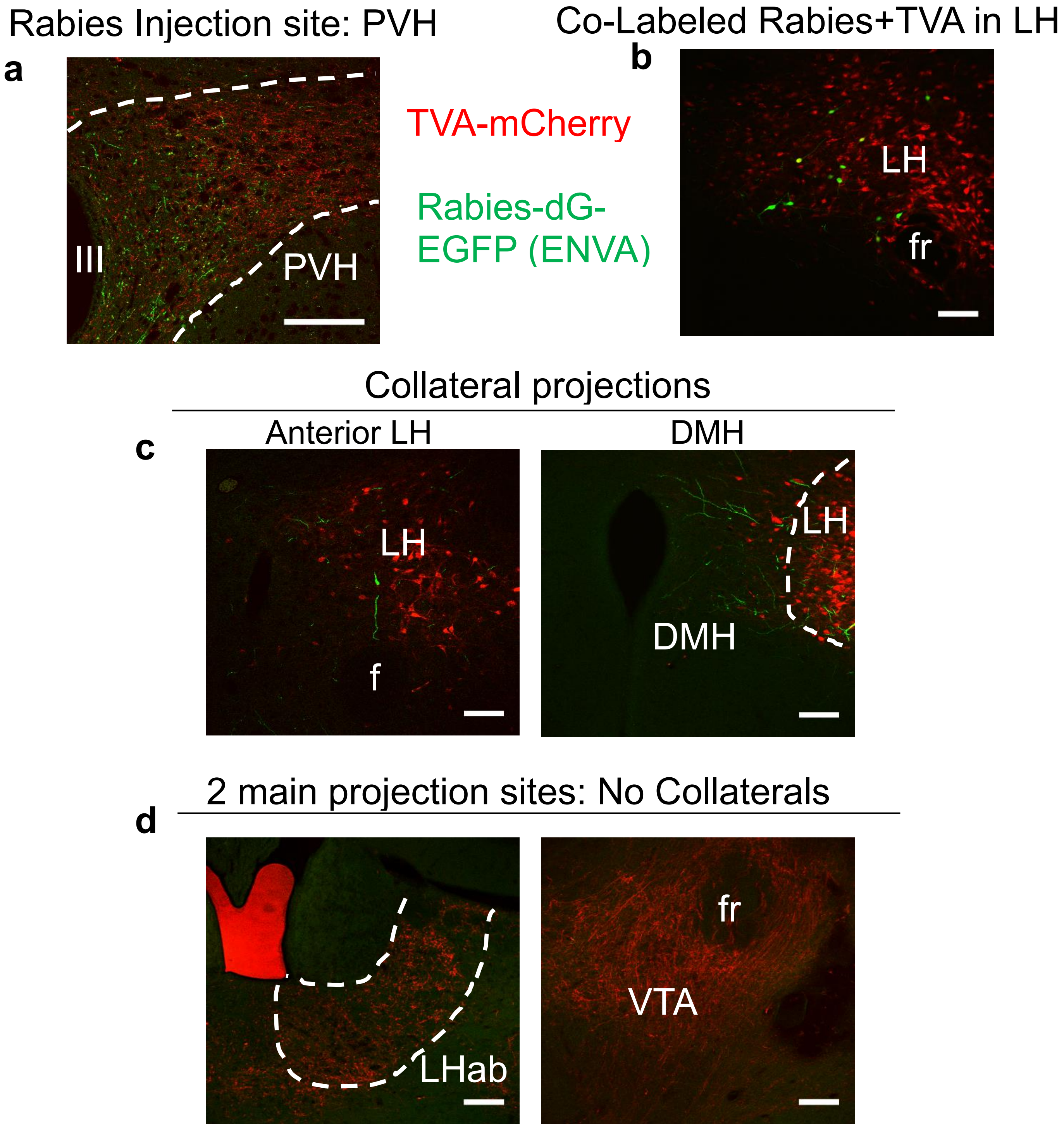

Supplementary Figure 14 PVH projecting LH neurons do not send collaterals to lateral habenula or VTA. Using *Pdx1-Cre* mice, we performed rabies terminal mapping study to assess the degree of collateralization of LH→PVH to other structures. Using the same protocol as described in previous studies (Betley et. al. Cell 2013 and Garfield et. al. Nat. Neurosci. 2015), we first delivered AAV-FLEX-TVA-mCherry to the LH of *Pdx1-Cre* mice, and 4 weeks later, delivered pseudotyped rabies virus deltaG to the PVH. As shown in panels **a** and **d**, TVA-expressing fibers were found in all known LH projection sites including PVH, lateral habenula and VTA. Importantly, abundant GFP-expressing fibers were also found in the PVH (panel **a**), confirming successful delivery of rabies virus in the PVH. As expected, a subset of LH neurons expressed GFP (panel **b**). However, no apparent GFP-expressing fibers were found in either lateral habenula or VTA (panel **d**). Interestingly, rare GFP-expressing fibers were found in the DMH and LH (panel **c**). These results suggest that the PVH-projecting LH neurons send an insignificant amount of collaterals to other projection sites. fr: fornix; III: 3rd ventricle; PVH:paraventricular hypothalamus; LH: lateral hypothalamus; DMH: dorsomedial hypothalamus; Lhab: lateral habenula; VTA: ventral tegmental area.

Supplementary Table 1 Mouse models used in Figures

Figure number	Genotypes used	Number of mice used	Virus used; Target region; Location of cells recorded or Optical Fiber placement OR experiment description
1d and 1f	*Pdx1-Cre*	-For oEPSCs and oIPSCs: 9 Mice (120 cells);<br>-For oEPSCs+CNQX/APV: 5 mice (6 cells);<br>-For oIPSCs+GABAzine: 5 mice (9 cells)	AAV-FLEX-ChR2-EYFP; LH; PVH
1e	*Pdx1-Cre*	-For oEPSCs+TTX/4-AP: 2 mice (8 cells);<br>-For oIPSCs+TTX/4-AP: 2 mice (8 cells)	AAV-FLEX-ChR2-EYFP; LH; PVH
2a-d	*Pdx1-Cre*<br><br>*Pdx1-Cre::Vgat$^{flox/flox}$*	-*Pdx1-Cre*: 3 Mice<br><br>- *Pdx1-Cre::Vgat$^{flox/flox}$*: 4 mice	AAV-FLEX-ChR2-EYFP; LH; PVH
2g-i	*Pdx1-Cre*<br><br>*Pdx1-Cre::Vgat$^{flox/flox}$*	7 mice total; Combined data from *Pdx1-Cre* (n=3) and *Pdx1-Cre::Vgat$^{flox/flox}$* (n=4) groups	AAV-FLEX-ChR2-EYFP; LH; PVH
3a-b	*Pdx1-Cre*<br><br>*Pdx1-Cre::Vglut2$^{flox/flox}$*	-*Pdx1-Cre*: 8 Mice<br><br>- *Pdx1-Cre::Vglut2$^{flox/flox}$*: 7 mice	AAV-FLEX-ChR2-EYFP; LH; PVH
3c-e	*Pdx1-Cre*<br><br>*Pdx1-Cre::Vglut2$^{flox/flox}$*	-*Pdx1-Cre* (GFP control): 6 mice<br><br>-*Pdx1-Cre::Vglut2$^{flox/flox}$*: 4 mice	-*Pdx1-Cre* (GFP control): AAV-FLEX-EGFP; LH; PVH<br>-*Pdx1-Cre::Vglut2$^{flox/flox}$*: AAV-FLEX-ChR2-EYFP; LH; PVH
3g-h	*Pdx1-Cre*<br><br>*Pdx1-Cre::Vglut2$^{flox/flox}$*	-*Pdx1-Cre*: 4 mice<br><br>-*Pdx1-Cre::Vglut2$^{flox/flox}$*: 3 mice	AAV-FLEX-ChR2-EYFP; LH; PVH
4a (included in 4c)		-For oEPSCs: 1 Mouse (12 cells)	AAV-FLEX-ChR2-EYFP; LH; PVH

	Pdx1-Cre::γ2$^{flox/flox}$	-For oIPSCs: 1 Mouse (12 cells)	
4b (included in 4c)	*Pdx1-Cre::Sim1-Cre::γ2$^{flox/flox}$*	-For oEPSCs: 1 Mouse (12 cells)<br>-For oIPSCs: 1 Mouse (12 cells)	AAV-FLEX-ChR2-EYFP; LH; PVH
4d	*Pdx1-Cre::γ2$^{flox/flox}$*<br><br>*Pdx1-Cre::Sim1-Cre::γ2$^{flox/flox}$*	-*Pdx1-Cre::γ2$^{flox/flox}$*: 7 mice<br><br>-*Pdx1-Cre::Sim1-Cre::γ2$^{flox/flox}$*: 6 mice	AAV-FLEX-ChR2-EYFP; LH; PVH
4g	*Pdx1-Cre::Vgat$^{flox/flox}$*	3 mice	AAV-FLEX-ChR2-EYFP; LH; PVH
5c-e	*Pdx1-Cre::Vglut2$^{flox/flox}$*	3 mice	AAV-FLEX-eArchT3.0-EGFP; LH; PVH
5g-i	*Pdx1-Cre::Vgat$^{flox/flox}$*	5 mice	AAV-FLEX-eArchT3.0-EGFP; LH; PVH
6b-c	*Pdx1-Cre::Vgat$^{flox/flox}$*	5 mice	AAV-FLEX-ChR2-EYFP; LH; PVH
6e	*Pdx1-Cre::Vglut2$^{flox/flox}$*	3 mice	AAV-FLEX-ChR2-EYFP; LH; PVH
6f	*Pdx1-Cre::Vglut2$^{flox/flox}$*	5 mice	AAV-FLEX-ChR2-EYFP; LH; PVH
7c-e	*Sim1-Cre*<br><br>*Sim1-Cre::Vglut2$^{flox/flox}$*	- *Sim1-Cre*: 4 mice<br><br>-*Sim1-Cre::Vglut2$^{flox/flox}$*: 4 mice	AAV-FLEX-ChR2-EYFP; PVH; PVH
7f	*Sim1-Cre*	1 mouse (5 cells)	AAV-FLEX-iC++-EYFP; PVH; PVH
7g-i	*Sim1-Cre*	-*Sim1-Cre* (GFP control): 4 mice<br><br>-*Sim1-Cre* (iC++ group): 6 mice	-*Sim1-Cre* (GFP control): AAV-FLEX-EGFP; PVH; PVH<br><br>-*Sim1-Cre* (iC++ group): AAV-FLEX-iC++-EYFP; PVH; PVH
Suppl. Fig. 1a-h	*Pdx1-Cre::Ai9*	1 representative mouse brain	N/A
Suppl. Fig. 1i-k	wild-type	1 representative mouse brain	*Vgat* and *Vglut2 in situ* hybridization
Suppl. Fig. 3a-b	*Pdx1-Cre::Vgat$^{flox/flox}$*	3 mice (25 cells)	AAV-FLEX-ChR2-EYFP; LH; PVH

Suppl. Fig. 3c-d	*Pdx1-Cre::Vglut2*$^{flox/flox}$	1 mouse (12 cells)	AAV-FLEX-ChR2-EYFP; LH; PVH
Suppl. Fig. 4e-h	*Pdx1-Cre*	4 mice	AAV-FLEX-EGFP; LH; PVH
Suppl. Fig. 6a	*Vglut2*$^{flox/flox}$	1 representative mouse brain	*Vglut2 in situ* hybridization
Suppl. Fig. 6b	*Pdx1-Cre::Vglut2*$^{flox/flox}$	1 representative mouse brain	*Vglut2 in situ* hybridization
Suppl. Fig. 6c	*Pdx1-Cre*<br><br>*Pdx1-Cre::Vglut2*$^{flox/flox}$	-*Pdx1-Cre*: 4 mice<br><br>-*Pdx1-Cre::Vglut2*$^{flox/flox}$: 3 mice	AAV-FLEX-ChR2-EYFP; LH; PVH
Suppl. Fig. 7a-b	*Sim1-Cre*<br><br>*Sim1-Cre::γ2*$^{flox/flox}$	-*Sim1-Cre*: 17 cells from 2 mice<br><br>-*Sim1-Cre::γ2*$^{flox/flox}$: 18 cells from 2 mice	*Ex-vivo* Loose-patch recordings
Suppl. Fig. 9	*Pdx1-Cre::Sim1-Cre::Ai9*	1 representative mouse brain	AAV-FLEX-Synaptophysin-GFP; LH/for anterograde tracing
Suppl. Fig. 10b	*Pdx1-Cre*	3 mice (4 cells)	AAV-FLEX-ChR2-EYFP/AAV-FLEX-eArchT3.0-EGFP; LH; PVH
Suppl. Fig. 10c	*Pdx1-Cre::Vglut2*$^{flox/flox}$	3 mice	AAV-FLEX-eArchT3.0-EGFP; LH; PVH
Suppl. Fig. 10e	*Pdx1-Cre*	1 mouse (2 cells)	AAV-FLEX-ChR2-EYFP/AAV-FLEX-eArchT3.0-EGFP; LH; PVH
Suppl. Fig. 11e	*Pdx1-Cre::Vglut2*$^{flox/flox}$	3 mice	AAV-FLEX-ChR2-EYFP; LH; PVH
Suppl. Fig. 12f	*Sim1-Cre*	1 mouse (3 cells)	AAV-FLEX-ChR2-EYFP; PVH; PVH
Suppl. Fig. 13c	*Sim1-Cre*	3 mice	AAV-FLEX-EGFP; PVH; PVH
