# Supplementary material for: A neural basis for antagonistic control of feeding and compulsive behaviors
Source: Nat Commun. 2018 Jan 4;9:52. doi: 10.1038/s41467-017-02534-9 (PMC5754347; doi:10.1038/s41467-017-02534-9)
Supplement: Supplementary file 2 — Description of Additional Supplementary Information [file 41467_2017_2534_MOESM2_ESM.pdf]

Description of Additional Supplementary Files

File Name: Supplementary Movie 1
Description: Video showing voracious feeding upon LH$^{Pdx1\text{-}ChR2}$→PVH photostimulation with 5Hz, 100 ms light pulses. Notice fast onset to feeding shortly after the light is turned on.

File Name: Supplementary Movie 2
Description: Video showing repetitive licking of cage upon LH$^{Pdx1\text{-}ChR2}$→PVH photostimulation in the absence of food.

File Name: Supplementary Movie 3
Description: Repetitive self-grooming is induced by photostimulation of the LH$^{Pdx1\text{-}ChR2}$→PVH circuit with 5Hz, 10 ms pulses of blue light.

File Name: Supplementary Movie 4
Description: Water spray strongly causes repetitive grooming, a behavior that is abruptly suppressed upon photostimulation of GABAergic LH$^{Pdx1\text{-}ChR2}$→PVH circuit in exchange for feeding. *Pdx1-Cre::Vglut2*$^{flox/flox}$ mice were used to target the non-glutmatergic (i.e., GABAergic) component of the circuit.

File Name: Supplementary Movie 5
Description: Video showing photostimulation of PVH$^{Sim1\text{-}ChR2}$ neurons abruptly stops ongoing feeding and quickly promotes grooming in a mouse fasted ~24h.

File Name: Supplementary Movie 6
Description: Photostimulation of non-glutamatergic neurons in PVH (using *Sim1-Cre::Vglut2*$^{flox/flox}$ mice) fails to suppress feeding and promote grooming during the fasted state. Notice mice will continue feeding regardless of light epoch.
